# Characterization of aquatic clade 2 and 3 *Campylobacter coli* isolates from Slovenia reveals admixture with other *Campylobacter* species

**DOI:** 10.1186/s12866-025-04042-z

**Published:** 2025-05-24

**Authors:** Stephan Lorenzen, Anastasia-Lisa Dieckmann, Sonja Smole Možina, Katja Zelenik, Pauline Marquardt, Aljoscha Tersteegen, Achim J. Kaasch, Wolfgang Bohne, Andreas Erich Zautner

**Affiliations:** 1 Bioinformatics Group, Bernhard Nocht Institute for Tropical Medicine, Hamburg, Germany; 2 Institute for Medical Microbiology and Virology, University Medical Center Göttingen, Göttingen, Germany; 3 Department of Food Science and Technology, Biotechnical Faculty, University of Ljubljana, Ljubljana, Slovenia; 4 Center for Microbiological Analysis of Food, Water and Other Environmental Samples, National Laboratory of Health, Environment and Food, Maribor, Slovenia; 5 Institute of Medical Microbiology and Hospital Hygiene, Medical Faculty, Otto von Guericke University Magdeburg, Magdeburg, Germany

**Keywords:** *Campylobacter coli*, Clade 2, Clade 3, Introgression, *Campylobacter lari *admixture, *Campylobacter jejuni *admixture

## Abstract

*Campylobacter coli*, a significant foodborne pathogen, has undergone extensive genetic exchange with its close relative, *Campylobacter jejuni*, leading to the emergence of three distinct clades. While clade 1 strains are commonly isolated from clinical and agricultural sources, clades 2 and 3 are primarily found in aquatic environments. This study aimed to enhance our understanding of *C. coli* clade 2 and 3 isolates through genomic and phenotypic characterization. A total of 48 surface water samples were collected from 19 different water bodies throughout Slovenia, and eleven *Campylobacter* isolates initially identified as *C. coli* from clades 2 and 3 were cultured. Whole genome sequencing was then performed on these isolates. Phylogenetic analysis was conducted using core genome multilocus sequence typing (cgMLST) and k-mer analysis. Phenotypic characterization included growth analysis, autoagglutination, biofilm formation, motility, antimicrobial susceptibility, water survival, and metabolic profiling. Genomic analysis revealed significant admixture with other *Campylobacter* species in the clade 2 and 3 isolates. One isolate was found to represent a new species related to *C. coli*. Besides *C. jejuni* and *C. lari*, this novel species appears to have contributed to introgression in the *C. coli* clades 2 and 3 isolates. Phenotypic characterization demonstrated diverse growth patterns, motility, autoagglutination abilities, and biofilm formation among the isolates. This study provides new insights into the genetic diversity and phenotypic characteristics of aquatic *C. coli* clade 2 and 3 isolates from Slovenia. The observed admixture with other *Campylobacter* species highlights the complex evolutionary history of these environmental strains and underscores the importance of continued surveillance and characterization of *Campylobacter* isolates from diverse ecological niches.

**Supplementary Information:**

The online version contains supplementary material available at 10.1186/s12866-025-04042-z.

## Introduction

*Campylobacter coli*, along with *Campylobacter jejuni*, is one of the most prevalent bacterial foodborne pathogens. It has undergone significant genetic exchange with its close relative, *C. jejuni*, resulting in the emergence of three distinct clades of *C. coli*, each exhibiting unique genetic and phenotypic characteristics [1]. This process of genetic introgression, which involves the transfer of genetic material between species through hybridization and backcrossing, has played a crucial role in shaping the genome of *C. coli* and *C. jejuni* [2, 3]. The mechanisms underlying horizontal gene transfer within the genus *Campylobacter* are still only partially understood. They involve, on one hand, natural transformation and uptake of free DNA, conjugative gene transfer, phage transduction, and genomic rearrangements. On the other hand, barriers to horizontal gene transfer must be overcome, such as CRISPR-Cas systems, periplasmic nucleases, and methylation-dependent DNA recognition/restriction modification (RM) systems [4–6].

*C. coli* strains are classified into three main clades. Clade 1 *C. coli* strains are most frequently isolated from stool samples of patients suffering from diarrhea, as well as from farm animals. These strains experience high levels of both intraclade and interspecies genetic exchange. In contrast, strains from the distinct clades 2 and 3 are commonly isolated from aquatic environments and waterfowl. Isolates from these clades undergo significantly less genetic exchange, occurring 5 to 10 times less frequently compared to *C. jejuni* [7–9]. Research by Sheppard and colleagues has shown that there is a significant ongoing genetic exchange between *C. coli* and *C. jejuni*, which may even lead to the convergence of these species and the formation of mosaic alleles [1]. These introgression events are especially prominent in agricultural environments, indicating an ongoing process that may be driven by selective pressures in these contexts [2]. The development of reference pan-genomes for *Campylobacter* species has yielded valuable insights into genetic diversity and potential epidemiological markers within the genus. Méric and colleagues employed this pan-genome approach to identify novel epidemiological markers in the accessory genome of pathogenic *Campylobacter* strains, aiding in the identification of specific populations (clades/sequence types) [10].

Due to the very limited number of phenotypically characterized *C. coli* clade 2 and 3 isolates and their representation from specific geographic regions e.g. Sweden [11], this study aims to enhance the understanding of *C. coli* clade 2 and 3 isolates by integrating genomic analyses with phenotypic characterization. In the analysis of surface water samples from different areas of Slovenia, 11 *Campylobacter* isolates were cultured, which were initially identified as *C. coli* of clades 2 and 3 through 7-gene MLST. Closed genomes were generated from these isolates using whole genome sequencing (WGS), and their phylogeny and gene introgression were examined through core genome MLST (cgMLST) and k-mer analysis. One isolate was found to represent a new species related to *C. coli*. There are indications that this species has contributed to introgression in the *C. coli* clades 2 and 3 isolates. Additionally, these isolates were phenotypically characterized regarding their growth curves, autoagglutination, biofilm formation, motility, antimicrobial susceptibility, water survival, and metabolism.

## Materials and methods

### Study site, bacterial culture, species identification

A total of 48 surface water samples were collected from 19 water bodies across the Styrian, Central, and Classical Karst regions of Slovenia. Primary isolation from water samples was performed following the ISO 17995 method [12]. A 1.0 L sample was filtered through multiple membrane filters with a pore size of 0.45 μm (cellulose nitrate filters, Whatman, Maidstone, Kent, United Kingdom). The filters were transferred into 100 mL of Preston broth (Nutrient Broth No. 2 & *Campylobacter* selective supplement & *Campylobacter* growth supplement; Thermo Fisher Scientific Inc., Waltham, Massachusetts, USA) and 100 mL of Bolton broth (Thermo Fisher Scientific Inc., Waltham, Massachusetts, USA) and incubated at 37 °C in a microaerobic atmosphere (using jars and Genbox microaer generators, bioMérieux, Nürtingen, Germany) for 48 h.

After incubation, the inocula were streaked onto modified charcoal cefoperazone deoxycholate agar plates (mCCDA, Thermo Fisher Scientific Inc., Waltham, Massachusetts, USA) and incubated at 41.5 °C in a microaerobic atmosphere for 24 to 48 h. Suspected colonies were subcultured onto two Columbia agar plates supplemented with 5% sheep blood (bioMérieux, Nürtingen, Germany). One plate was incubated at 41.5 °C in a modified atmosphere, while the other was incubated aerobically at 41.5 °C. Cultures that did not grow aerobically were further examined using phase-contrast microscopy (DM 2500, Leica, Wetzlar, Germany). If *Campylobacter*-suspicious motile slender rods with spiral morphology were observed they were sub-cultured for DNA Isolation, and preliminary species identification was performed using multiplex PCR as described by Wang and colleagues [13]. Species confirmation was conducted using the MALDI Biotyper Autoflex III System (Bruker Daltonics, Bremen, Germany) as previously described [14].

### Multilocus sequence typing (MLST)

For an initial clade assignment the sequence type (ST) was established using the standard amplification and sequencing primers as previously reported (https://pubmlst.org/campylobacter/info/primers.shtml). The cycling conditions included an initial denaturation at 94 °C for 1 min, followed by 35 cycles consisting of denaturation at 94 °C for 2 min, annealing at 50 °C for 1 min, and extension at 72 °C for 1 min. The process concluded with a final elongation step at 72 °C for 5 min [15]. Amplicons of the seven genes included in the *C. coli*/*C. jejuni* MLST scheme were sent for sequencing to Microsynth Seqlab (Göttingen, Germany), utilizing 10 pmol of the respective sequencing primer. The Mega X software for Linux was employed to calculate an MLST-based evolutionary history using the neighbor-joining method [16, 17]. Evolutionary distances were computed using the Maximum Composite Likelihood method [18].

### Genome sequencing

Isolation of bacterial DNA was performed via the cetyltrimethylammonium bromide (CTAB) method as detailed in Bechman et al. [19]. Library preparation for Illumina paired-end sequencing was performed with the NEBNext^®^ UltraTM II FS DNA Library Prep Kit for Illumina #E6177 (New England Biolabs GmbH, Frankfurt am Main, Germany). Libraries were barcoded using the NEBNext^®^ MultiplexOligos for Illumina^®^ 96 Unique Dual Index Primer Pairs #E6440S/L (New England Biolabs GmbH, Frankfurt am Main, Germany) and sequenced using the MiSeq Reagent Kit v2 (500-cycles, Illumina) as described by the manufacturer. Barcoded libraries for Oxford Nanopore long-read sequencing were prepared using the Rapid Barcoding Kit 96 (SQK-RBK110.96) according to the manufacturer’s instructions and sequenced on aR9.4.1 flow cell (FLO-MIN106) on the MinION platform (Oxford Nanopore technologies ltd., Oxford, United Kingdom). Illumina paired-end reads were preprocessed using fastp (https://github.com/OpenGene/fastp, v0.23.2) and filtlong (parameters: --min_length 1000 --keep_percent 95, https://github.com/rrwick/Filtlong, v0.2.1). Genomes were assembled by unicycler v0.5.0 using the standard parameters for a hybrid assembly (4 threads, modus “normal”). Assembly quality was assessed with QUAST v5.2.0 [20]. The assemblies were annotated using the online NCBI Prokaryotic Genome Annotation Pipeline (PGAP) accessed as indicated in each individual GenBank entry [21–23]. A taxonomy check was performed using mash v2.3 [24].

### Bioinformatics

Core genomes were calculated using Roary version 3.13 [25] utilizing the gnomes of the 11 test isolates, the 8 genomes published by Nilson et al. [11] as well as the reference strains *C. lari* RM2100^T^, *C. novaezeelandieae* W441b^T^, *C. jejuni* NCTC 11,168, *C. jejuni* 81–176, *C. coli* BFR-CA-9557, *C. coli* RM2228, and *C. coli* 76339, which will be plotted in the later phylogeny as input. Roary was used with parameters -r -z -e and the identity threshold was specified using the parameter -i. RAxML (Randomized Axelerated Maximum Likelihood version 8.2.12) was used to perform maximum likelihood-based phylogenetic analysis [26] with parameters “-f a -# autoMRE”. The GTR (General Time Reversible) model was applied for nucleotide substitution, while the CAT model approximated rate heterogeneity across sites. Sequences with ≥ 95% identity were clustered, and only alignment positions present in at least 90% of the sequences were considered in the analysis.

To examine introgression, the genomes were split in overlapping 1,000 nt pieces with 500 nt distance from each other and the fragments were aligned to the nt database using blastn version 2.16 [27]. The taxonomic annotation of the best hit was mapped to species level using inhouse tools (Supplementary Material 1). Heatmaps were generated to visualize the identity level, calculated as the number of identical nucleotides divided by the length of the input sequence, using R version 4.4.1 [28].

### Phenotypic assays

#### Bacterial culture

Bacteria were cultured for 17 to 18 h on Columbia agar plates supplemented with 5% sheep blood (COS, bioMérieux, Germany) at 37 °C under microaerophilic conditions. The microaerophilic atmosphere was generated using the Gas Pak™ EZ Campy Container System by BD (Franklin Lakes, NJ, USA) along with an anaerobic jar for incubation.

### Growth analysis and colony morphology assessment

Bacteria were harvested from COS agar plates (grown for 17–18 h under microaerophilic conditions) and resuspended into 10 mL of MH Broth to a starting OD_600nm_ of 0.05. These bacterial suspensions were then incubated at 37 °C and 150 rpm in microaerophilic conditions and growth measurements were simultaneously taken for approximately 48 h using the Certomat^®^ U shaker by B. Braun Biotech International GmbH (Melsungen, Germany), the cell growth quantifier from aquila biolabs GmbH (Baesweiler, Germany) and the CGQuant 8.2.2. software program. The growth measurements were taken for each strain in biological triplicates. The backscatter measurements were converted to OD_600nm_ readings using the CGQuant 8.2.2. calibration program. The calibration was done as per manufacturer instructions using *C. jejuni* NCTC 11,168 as reference strain. The colony morphology after 24 h at 37 °C on the COS agar plates was assessed according to the criteria established by Sousa and colleagues [29, 30].

### Motility

Motility assays were performed as described by Tareen et al. (2010) with minor modifications [35]. Bacteria were cultured in MH Broth for 16 h at 37 °C and 150 rpm. The bacterial suspension was then adjusted to an OD_600nm_ of 0.025 and these suspensions were then stabbed into 0.4% MH soft agar plates with a 1 µL inoculation loop. The plates were incubated at 37 °C under microaerophilic conditions for 36 h, after which the diameters of the swarming zones were measured with a ruler. Motility experiments were performed in technical duplicates and biological triplicates.

### Autoagglutination assay

Autoagglutination assays were performed as described by Misawa and Blaser (2000) [31]. Bacteria were resuspended from COS agar plates in PBS (pH 7.4) and adjusted to an OD_600nm_ of 1. The bacterial suspensions were incubated in 2 mL volumes for 24 h at 37 °C under microaerophilic conditions without shaking. After incubation, 1 mL of the supernatant was carefully removed and the OD_600nm_ was measured. The experiments were performed in technical and biological triplicates.

### Biofilm formation

The biofilm assays were performed as described by Reeser et al. (2007) with some modifications [32]. Bacteria were harvested from COS agar plates and resuspended in MH Broth to an OD_600nm_ of 0.05. The bacterial suspensions were then added in 100 µL volumes into the wells of a 96-well plate and the plate was incubated for 48 h at 37 °C under microaerophilic conditions. After incubation, the broth was removed from each well and the plate was dried for 30 min at 60 °C. The wells were stained with 100 µL of 0.1% crystal violet solution for 15 min at room temperature, after which the unbound solution was removed. The wells were then washed two times with 200 µL of water and the plate was again dried for 15 min at 60 °C. The bound crystal violet was then suspended into solution by adding 100 µL of an 80% ethanol-20% acetone solution. An amount of 80 µL of the suspensions were then added into a fresh 96-well plate. The plate was read at an absorbance of 570 nm with a microplate reader to quantify the biofilm formation in each well. The experiments were done in technical quadruplets and biological triplicates.

### Water survival

Deionized water was sterile filtered using 0.2 μm sterile filters and autoclaved. Bacteria were harvested from COS plates, added to 5 mL volumes of the filtered and autoclaved water to a final OD_600nm_ of 0.05 (corresponding to a concentration of approximately 2 × 10^8^ CFU/ml) and incubated in a fridge at 4 °C under aerobic conditions. Samples for viable counts were taken at the start of the experiments and on days 2 and 4. These were immediately plated out on COS agar plates in serial dilutions and incubated at 37 °C in microaerophilic conditions for 48 h. The experiment was performed in biological triplicates.

### Statistical analyses

Statistically significant differences between the phenotypic behaviors of the individual bacterial strains were assessed using the unpaired t test. A p-value of less than 0.05 was considered significant. Statistical significant differences between clades were assessed using a linear mixed-effects model 2 (Supplementary Material 2) using the “lmerTest” package in R version 4.4.3. The model included the clade structure as a fixed effect and strain identity as a random effect to account for biological replicates. P-values were calculated using Satterthwaite’s approximation.

### Basic metabolic profiling

To provide an initial metabolic profile of the studied isolates, an analysis was conducted using the Analytical Profile Index for *Campylobacter* (API Campy) according to the manufacturer’s instructions (Biomerieux, Nürtingen, Germany). Each isolate was tested twice, and in cases of discrepancies, a third test was performed.

## Results

### Isolation, species identification, phylogeny, and clade designation

A total of 48 water samples were analyzed, resulting in the culture of 25 phenotypically identified *Campylobacter* isolates. Using the multiplex PCR method developed by Wang and colleagues, all of these isolates were preliminary identified to be *C. coli*.

**Fig. 1:**
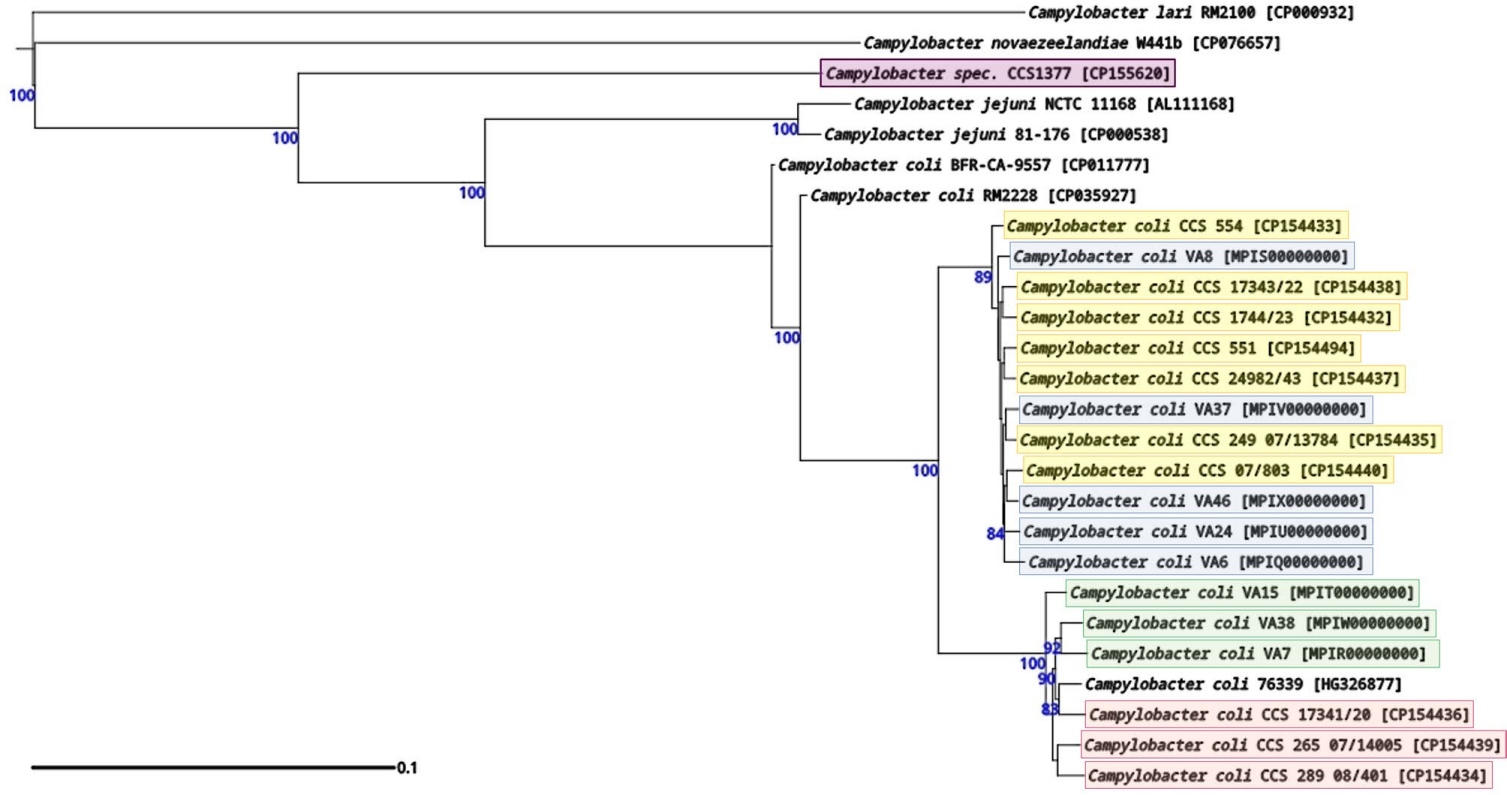
Phylogenetic dendrogram of the 11 analyzed Slovenian *Campylobacter* water isolates, including reference sequences from NCBI GenBank and from the study conducted by Nilsson and colleagues [11]. This phylogenetic tree was generated using RAxML version 8.2.12 with the following parameters: a sequence identity threshold of 95%, an alignment position presence threshold of 90%, and the GTR evolutionary model with a CAT approximation for rate heterogeneity. GenBank accession numbers for each strain are provided in square brackets. Study isolates were marked with yellow boxes for clade 2 and red boxes for clade 3, while the isolates characterized by Nilsson and colleagues were represented with blue boxes for clade 2 and green boxes for clade 3. Reference strains were depicted without any boxes. Bootstrap values (≥ 60%) based on 1,000 replications are indicated in blue at the nodes. The branch lengths are scaled in terms of evolutionary distance according to the bar.

In the preliminary genotyping of the *C. coli* isolates using the seven-gene MLST (Supplementary Fig. 1, Table 1) approach, seven isolates were classified into clade 2, three isolates into clade 3, and the remaining fourteen isolates were assigned to clade 1. The isolate CCS1377, with the ST7908, branched off at the base of the clade 2 subtree. The eleven non-clade 1 isolates were completely genome sequenced. The core-genome MLST-based phylogeny (Fig. 1), which included the aforementioned Swedish clade 2/3 isolates characterized by Nilsson and coworkers [11] along with several reference strain genomes, confirmed the clade assignments for the seven clade 2 and three clade 3 isolates. In contrast, the isolate CCS1377 was phylogenetically positioned between *Campylobacter novaezeelandiae* and the *C. jejuni*/*C. coli* subtree. An analysis of the Average Nucleotide Identity (ANI) for the genomes deposited in NCBI revealed a maximum value of 82.133 (query coverage: 32.9; subject coverage: 31.4) in relation to *Campylobacter coli* NCTC11366^T^ (GenBank accession: GCA_900446355.1). This suggests that the isolate CCS1377 may represent a new microbial species.

**Table 1:** Basic data of the *Campylobacter* water isolates included in the study (Bioproject PRJNA1105365)

No.	Isolate (DSM No.)	year of isolation	MLST-ST	clade	Genome size (Mbp)	Biosample	GenBank Accession	MALDI-ToF score value
1.	CCS 24,982/43 (DSM 118014)	2006	7863	2	1.748	SAMN41105154	CP154437	2.10
2.	CCS 17,343/22 (DSM 118015)	2006	1981	2	1.747	SAMN41105164	CP154438	2.16
3.	CCS 07/803 (DSM 118016)	2007	7862	2	1.867	SAMN41105168	CP154440	2.03
4.	CCS 1744/23 (DSM 118019)	2006	5658	2	1.841	SAMN41105169	CP154432	2.06
5.	CCS 249 07/13,784 (DSM 102057)	2007	7859	2	1.860	SAMN05412813	CP154435	2.11
6.	CCS 554 (DSM 118028)	2008	2269	2	1.907	SAMN41105181	CP154433	2.14
7.	CCS 551 (DSM 118029)	2008	7854	2	1.820	SAMN41105183	CP154494	2.11
8.	CCS 17,341/20 (DSM 101855)	2006	7907	3	1.589	SAMN05003719	CP154436	1.75
9.	CCS 265 07/14,005 (DSM 118022)	2007	7909	3	1.608	SAMN41105170	CP154439	1.78
10.	CCS 289 08/401 (DSM 118025)	2008	7910	3	1.618	SAMN41105180	CP154434	1.88
11.	CCS 1377 (DSM 102056)	2008	7908	not applicable	1.853	SAMN04922415	CP155620	1.58 (no ID)

**Fig. 2:**
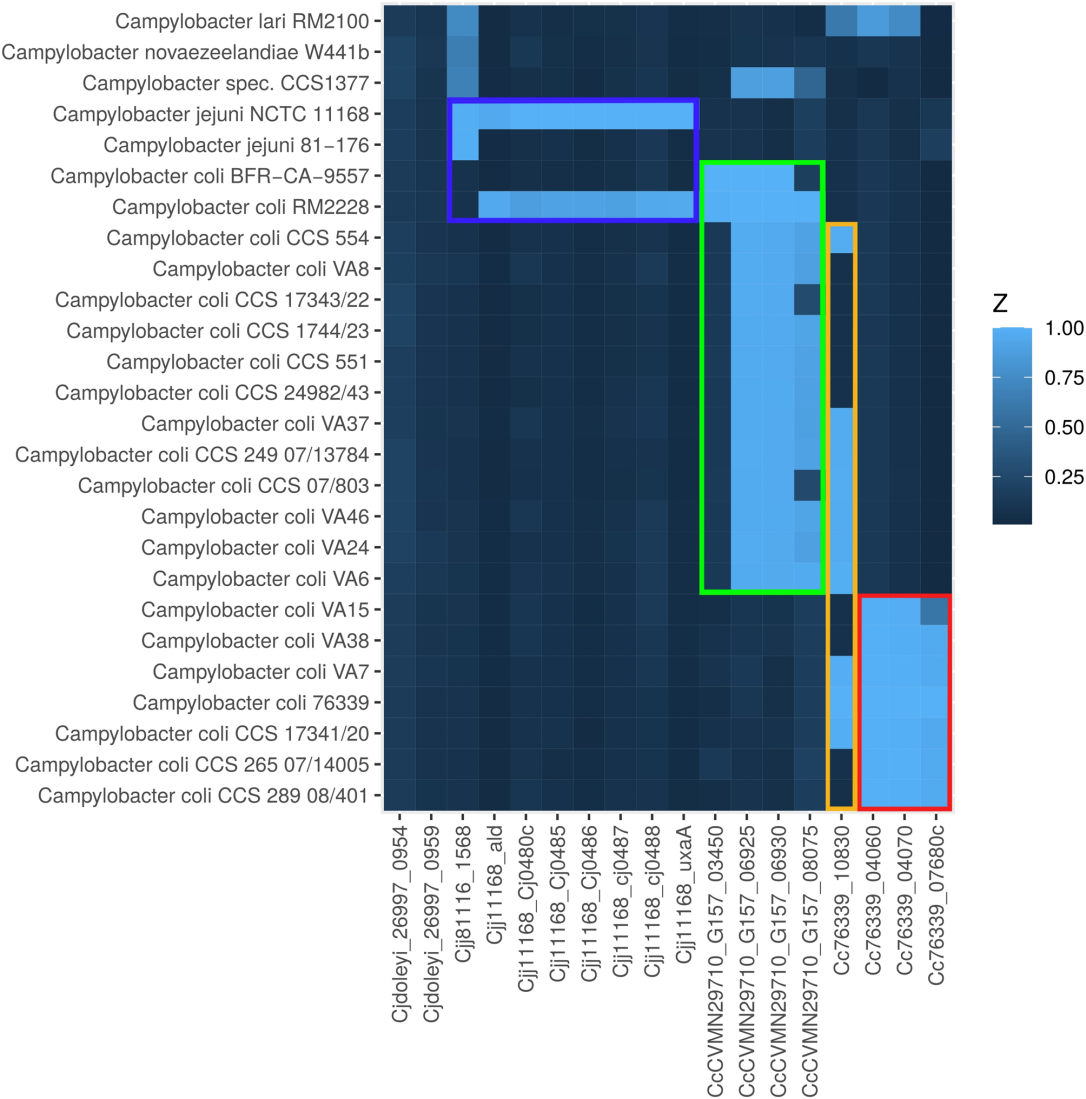
Heatmap indicating the presence of clade specific genes according to Méric et al. 2014. *Campylobacter* isolates (genomic sequences) are listed in phylogenetic order according to the dendrogram presented in Fig. 1. The columns represent 18 clade-specific genes identified through core-genome analyses by Méric and colleagues [10]. The color coding is as follows: red frame: clade 3, yellow frame: clades 2 and 3, green frame: clades 1 and 2, and blue frame: clade 1 and *C. jejuni*. The intensity of the blue color corresponds to the % identity from the BLAST analysis. Notably, *Campylobacter* sp. CCS1377 is positive for three clade 2-specific genes, while *C. lari* RM2100 is positive for two clade 3-specific genes and one gene specific to clades 2 and 3.

To further confirm the clade designation, these 11 Slovenian surface water isolates were examined alongside reference genomes for the presence of clade-specific genes as per Méric et al. [10]. The analysis (Fig. 2) confirmed the presence of three clade 3-specific genes in all presumed clade 3 isolates, as well as the presence of the clade 2/3-specific gene (Cc76339_10830) in 10 of the presumed clade 2 and 3 isolates. Additionally, three clade 1/2-specific genes were detected in the clade 2 isolates, supporting the previously established clade assignments. For isolate CCS1377, three clade 2-specific genes were also detected. Furthermore, the *C. lari* reference strain RM2100 was tested positive for two clade 3-specific genes and one clade 2/3-specific gene.

In accordance with these results, the clade 2 isolates could be reliably identified by MALDI-TOF MS (Table 1) as *C. coli*, with score values exceeding 2. In contrast, the clade 3 isolates yielded uncertain MALDI-TOF-IDs with score values around 1.8, and no identification was possible for CCS1377, which had a max. score value of 1.58 for *C. coli*.

### k-mer-analysis and admixture of other *Campylobacter* species

**Fig. 3:**
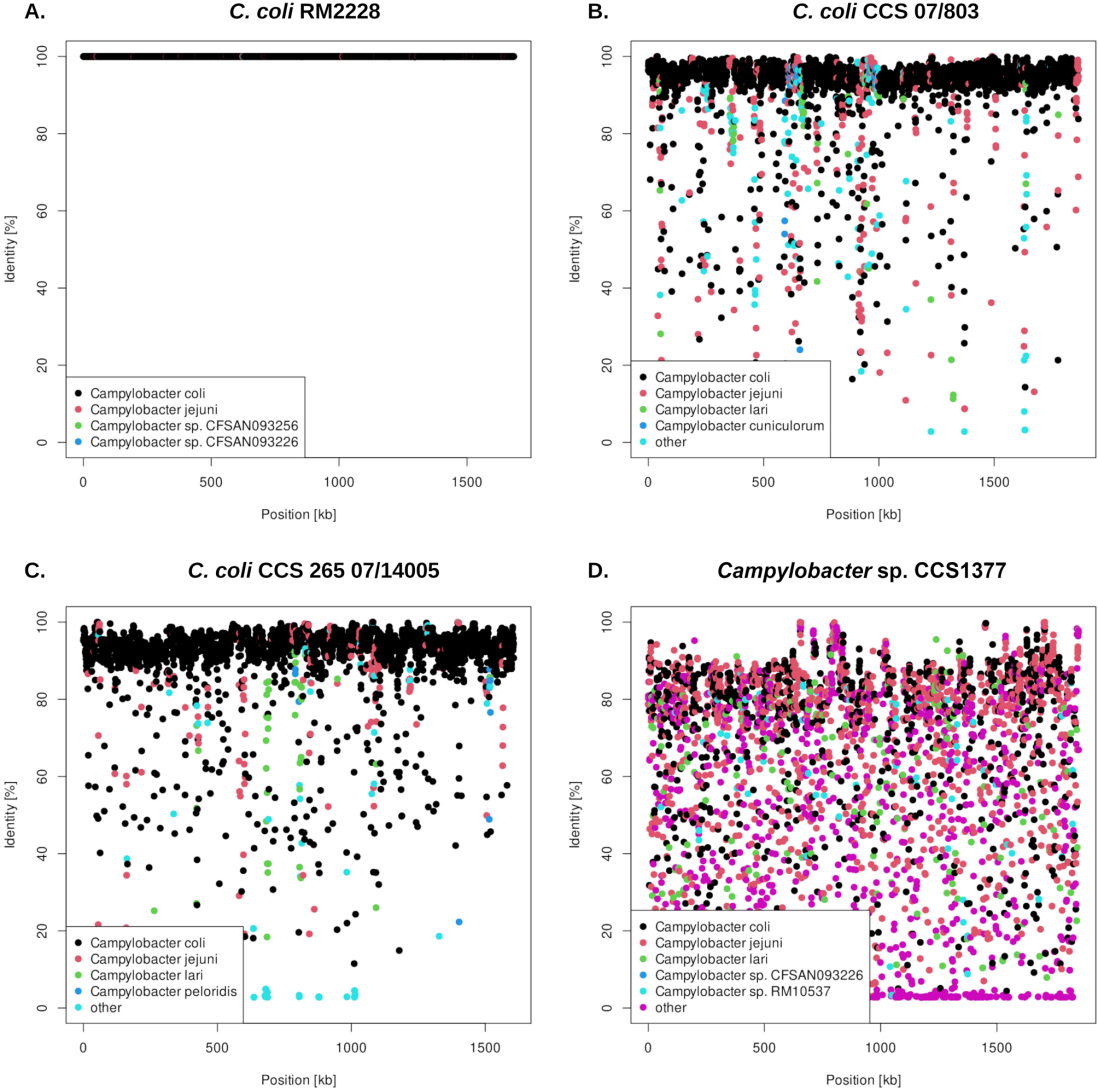
1,000-mer analysis (confetti-blots) of *C. coli* RM2228 (clade 1), *C. coli* CCS07/803 (clade 2), *C. coli* CCS265 07/14,005 (clade 3), and *Campylobacter* sp. CCS1377. The genomes of each *Campylobacter* isolate were in silico fragmented into 1,000 bp segments and subjected to BLAST analysis against the genomes in NCBI GenBank. Each segment is represented as a dot, with the color of each dot indicating the species of the second best hit. *C. coli* RM2228 (clade 1, Fig. 3A) shows nearly no admixture with other *Campylobacter* species, while *C. coli* CCS 07/803 (clade 2, Fig. 3B) displays a significant admixture of *C. jejuni* (9.45%) and *C. lari* (1.59%). A similar pattern is also observed in *C. coli* CCS265 07/14,005 (clade 3, Fig. 3C), which exhibits admixture with *C. jejuni* (5.70%) and *C. lari* (1.39%). In contrast, *Campylobacter* sp. CCS1377 exhibits a variable fragment matching pattern of 26.31% matching to *C. coli*, 32.81% to *C. jejuni*, 5.99% to *C. lari*, and 34.88% to other *Campylobacter* species, with a significantly lower sequence identity for each 1,000-mer.

In the context of the k-mer analysis, the genomes of each isolate were in silico fragmented into 1,000 bp segments (k = 1,000). These segments were individually subjected to a BLAST search to identify their second-best hits in NCBI GenBank (Since the genomes had already been uploaded to NCBI GenBank by that time, the top hit would refer to itself.). The results of this analysis are exemplified in confetti plots (Fig. 3). In contrast to the reference genome of the clade 1 isolate *C. coli* RM2228, which contains only a minimal admixture of approximately 1% *C. jejuni* genetic material (Fig. 3A; Table 2), the genomes of clade 2 and clade 3 exhibit significantly greater introgressions from other *Campylobacter* species. In the clade 2 genomes, the *C. jejuni* admixture ranges from 6.58 to 9.46%, while in the clade 3 genomes, the *C. jejuni* admixture is lower, ranging from 5.39 to 6.66% (Table 2; Fig. 3B and C) (Table 3).

**Table 2:** Percentage admixture of *C. coli*, *C. jejuni*, and *C. lari* in the 11 study isolates and reference strains

isolate	GenBank Accession	clade	admixture (%)			
			C. coli	C. jejuni	C. lari	other^1^
RM2100	CP000923	n.a	0.16	0.36	93.91	5.56
W441b	CP076657	n.a	9.97	36.00	3.66	50.37
CCS 1377	CP155620	n.a.	26.31	32.81	5.99	34.88
NCTC 11,168	AL111168	n.a	0.09	97.99	0.00	1.92
81–176	CP000538	n.a	0.06	96.53	0.00	3.40
BFR-CA-9557	CP011777	1	97.01	2.76	0.00	0.23
RM2228	CP035927	1	98.69	1.16	0.00	0.15
CCS 554	CP154433	2	85.15	9.46	0.92	4.47
VA8	MPIS00000000	2	87.55	7.98	0.65	3.82
CCS 17,343/22	CP154438	2	87.89	6.81	1.36	3.95
CCS 1744/23	CP154432	2	87.04	7.98	0.96	4.02
CCS 551	CP154494	2	86.22	8.34	0.94	4.50
CCS 24,982/43	CP154437	2	88.79	7.48	0.59	3.18
VA37	MPIV00000000	2	88.78	7.12	0.74	3.36
CCS 249 07/13,784	CP154435	2	85.33	9.32	1.46	3.89
CCS 07/803	CP154440	2	84.04	9.45	1.59	4.91
VA46	MPIX00000000	2	87.87	8.10	0.55	3.49
VA24	MPIU00000000	2	89.46	6.78	0.56	3.20
VA6	MPIQ00000000	2	90.12	6.58	0.44	2.87
VA15	MPIT00000000	3	90.48	6.65	0.33	2.54
VA38	MPIW00000000	3	90.48	6.65	0.43	2.45
VA7	MPIR00000000	3	91.41	6.66	0.31	1.63
76,339	HG326877	3	91.75	5.39	0.48	2.39
CCS 17,341/20	CP154436	3	90.94	6.59	0.38	2.09
CCS 265 07/14,005	CP154439	3	90.11	5.70	1.39	2.80
CCS 289 08/401	CP154434	3	91.81	5.73	0.31	2.15

^1^ the column “other” includes not only other species of the genus *Campylobacter* as well k-mers that could not be clearly assigned (This is in part a consequence that some sequences in NCBI GenBank are deposited under the species designation “*Campylobacter* sp.”)

**Table 3:** API results of the 11 study isolates, as well as the *C. coli* clade 1 and *C. jejuni* reference isolates

isolate	clade	URE	NIT	EST	HIP	GGT	TTC	PyrA	ArgA	AspA	PAL	H2S	GLU	SUT	NAL	CFZ	ACE	PROP	MLT	CIT	ERO	CAT	%
CCS 24,982/43	2	-	+	+	**-**	**-**	**+**	-	+	-	+	-	-	+	-	+	-	+	-	+	-	+	99.9
CCS 17,343/22	2	-	+	+	-	+	+	-	+	-	+	-	-	+	-	+	-	-	-	-	-	+	87.6
CCS 07/803	2	-	+	+	-	-	+	-	+	-	+	-	-	+	-	+	-	+	-	-	-	+	99.9
CCS 1744/23	2	-	+	+	-	+	+	-	+	-	+	-	-	+	-	+	-	+	-	+	-	+	99.7
CCS 249 07/13,784	2	-	+	+	-	+	+	-	+	-	+	-	-	+	-	+	-	+	-	+	-	+	99.7
CCS 554	2	-	+	+	-	-^1^	+	-	+	-	+	-	-	+	-	+	-	-	-	-	-	+	98.5
CCS 551	2	-	+	+	-	+	+	-	+	-	+	-	-	+	-	+	-	+	-	+	-	+	99.7
CCS 17,341/20	3	-	+	+	-	+	+	-	+	-	+	-	-	+	-	+	-	-	-	+	-	+	94.7
CCS 265 07/14,005	3	-	+	+	-	+	+	-	+	-	+	-	-	+	-	+	-	-	-	+	-	+	87.6
CCS 289 08/401	3	-	+	+	-	+	+	-	+	-	+	-	-	+	-	+	-	-	-	+	-	+	88.3
CCS 1377	n.a.	-	+	+	-	-	+	-	+	-	+	-	-	-	-	-	-	-	-	-	-	+	99.7^*^
BFR-CA-9557	1	-	+	+	-	-	+	-	+	-	+	-	-	+	-	+	-	-	-	-	-	+	98.5
RM2228	1	-	+	+	-	-	+	-	+	-	+	-	-	+	-	+	-	-	-	-	+	+	99.9
NCTC 11,168	n.a.	-	+	+	+	-	+	+	+	-	+	-	-	+	-	+	-	-	-	+	-	+	97.9
81–176	n.a.	-	+	+	+	+	+	+	+	-	+	-	-	+	-	+	-	-	-	-	-	+	99.9

^1^ 554 disrupted *ggt* gene ^*^ Identification as *Campylobacter lari*

^*^ Identification as *Campylobacter lari*

Following *C. jejuni* admixture, the second most prevalent admixture comes from *C. lari*, with proportions ranging from 0.44 to 1.59% in the clade 2 genomes and slightly lower percentages of 0.31–1.39% in the clade 3 genomes. The graphical representation of the k-mer analysis of the *Campylobacter* sp. CCS1377 genome (Fig. 3D) stands out as a figurative confetti plot, displaying an admixture of 26.31% from *C. coli*, 32.81% from *C. jejuni*, 5.99% from *C. lari*, and 34.88% from other *Campylobacter* species. It is also important to note that, in contrast to the three *C. coli* genomes, the sequence identity of the particular 1,000-mers often falls significantly below 90%. For the fragments that match *C. coli*, the average sequence identity is 71.1%. For those matching *C. jejuni*, the average is 68.7%, while fragments aligning with *C. lari* show an average sequence identity of 57.0%. Fragments that correspond to other *Campylobacter* species exhibit an average sequence identity of 44.7% (Table 2; Fig. 3D). However, BLAST search results in the “other” category often relate to genomes that are only classified as *Campylobacter* species in the NCBI GenBank database, without specifying the exact microbial species.

**Fig. 4:**
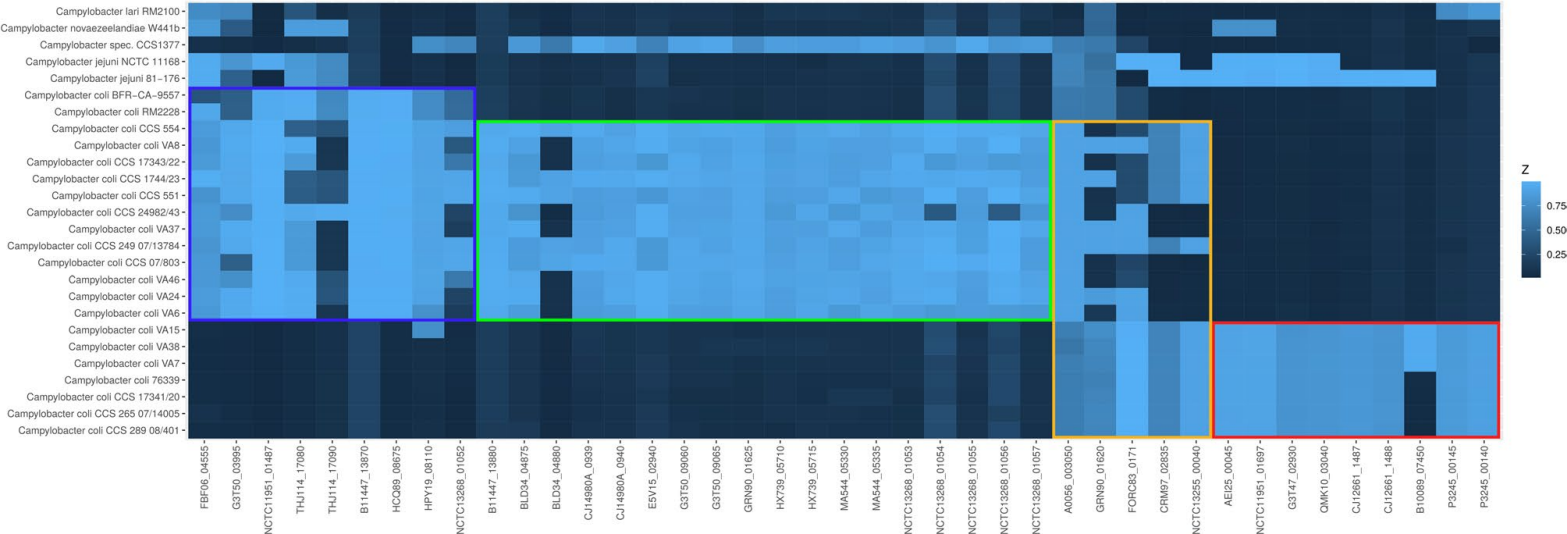
Heatmap indicating the presence of clade-specific genes introgressed from *C. jejuni*. The *Campylobacter* genomic sequences are organized in phylogenetic order based on the dendrogram presented in Fig. 1. The columns represent 41 clade-specific genes (indicated by their locus tag) identified through the 1,000-mer analysis as introgressed from *C. jejuni* (legend see Supplementary Table 1). The color coding is as follows: red frame: clade 3, yellow frame: clades 2 and 3, green frame: clade 2, and blue frame: clade 1 and 2. The intensity of the blue color corresponds to the % identity from the BLAST analysis. Notably, *Campylobacter* CSS1377 is positive for the majority of clade 2-specific genes

**Fig. 5:**
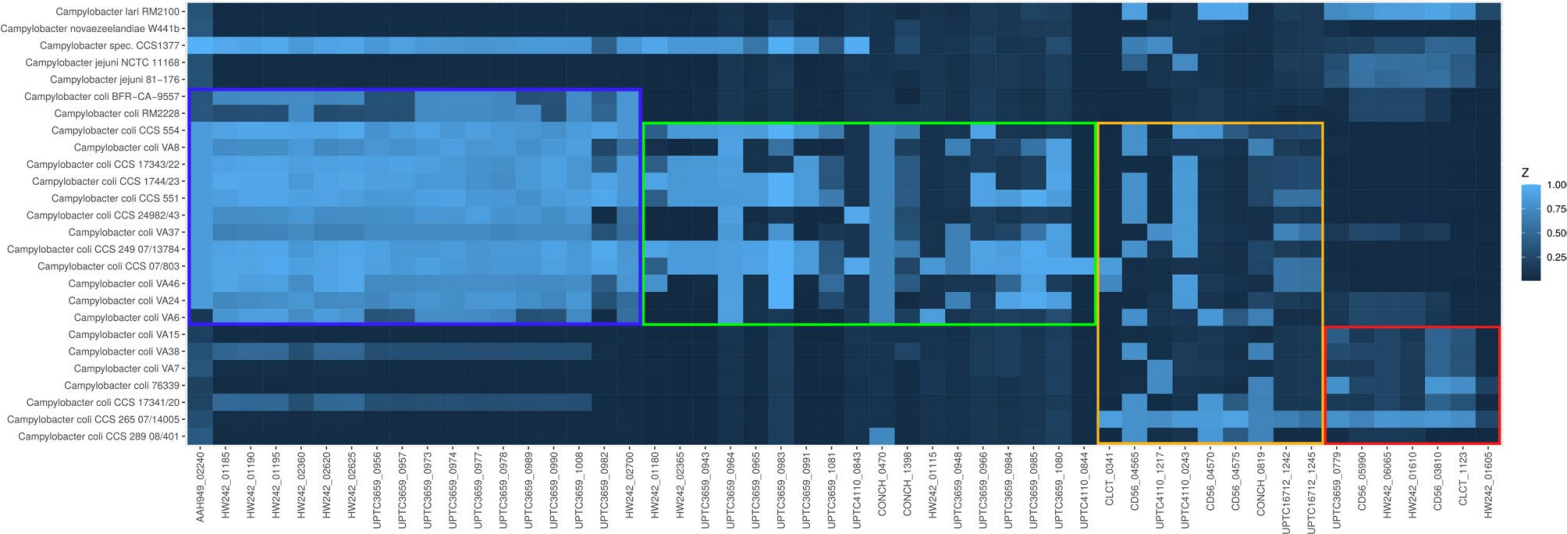
Heatmap indicating the presence of clade-specific genes introgressed from *C. lari*. The *Campylobacter* genomic sequences are organized in phylogenetic order based on the dendrogram presented in Fig. 1. The columns represent 52 clade-specific genes (indicated by their locus tag) identified through the 1,000-mer analysis as introgressed from *C. lari* (legend see Supplementary Table 2). The color coding is as follows: red frame: clade 3, yellow frame: clades 2 and 3, green frame: clade 2, and blue frame: clade 1 and 2. The intensity of the blue color corresponds to the % identity from the BLAST analysis. Notably, *Campylobacter* sp. CSS1377 is positive for the majority of clade 1 and 2-specific genes and ca. half of the clade 2-specific genes

To investigate whether there is a consensus among the introgressed genetic material, we compared the individual 1,000 bp fragments of each genome within a clade using BLAST. The remaining fragments were then assigned to genes through a BLAST search against the NCBI GenBank. The list of consensus genes for *C. jejuni* is presented in Supplementary Table 1, along with their presence in the analyzed genomes in heatmap Fig. 4. For the significantly smaller number of *C. lari* fragments, we omitted the initial reduction step of the consensus search. Instead, all fragments from the genomes with the maximum *C. lari* admixture for clade 2 (CCS 07/803) and clade 3 (CCS 265 07/14005) were assigned to their respective genes. Based on these genes, which are listed in Supplementary Table 2, a heatmap was also created to illustrate their presence in the examined genomes (Fig. 5).

Assigning functional roles to the majority of identified genes was challenging, as many of their presumed gene products were classified as hypothetical proteins. However, for the well-annotated genes, we have observed clear clade associations. Our analysis of the introgression of *C. jejuni* genes into *C. coli* genomes revealed significant patterns in the presence of enzyme and protein-encoding genes across the three clades of *C. coli*. This suggests potential metabolic and functional differences among these groups.

Clades 1 and 2 shared four enzymes, including D-lactate dehydrogenase, peptidase C39, YeeE/YedE family protein, and a small methyltransferase. The presence of D-lactate dehydrogenase suggests these clades may have enhanced capabilities for lactate metabolism, potentially providing an advantage in certain environmental niches, while the peptidase C39 might play a role in protein processing or degradation.

Clade 2 genomes exhibited unique enzyme genes, including methionyl-tRNA formyltransferase, UDP-4-amino-4,6-dideoxy-*N*-acetyl-beta-L-altrosamine *N*-acetyltransferase (*pseH*), and an outer membrane siderophore receptor. The presence of a methionyl-tRNA formyltransferase gene suggests that clade 2 may have distinct protein synthesis initiation mechanisms. The PseH enzyme is involved in protein glycosylation, which could affect cell surface properties and host interactions. The siderophore receptor indicates enhanced iron acquisition capabilities in this clade, potentially conferring a competitive advantage in iron-limited environments.

Both clades 2 and 3 possessed a citrate transporter and the gamma-glutamyltransferase (Ggt). The citrate transporter suggests these clades may have improved citrate utilization capabilities, potentially expanding their metabolic versatility. The presence of Ggt indicates enhanced glutamin/glutamat/glutathione metabolism of these isolates.

Clade 3 exhibited a unique set of enzymes, including cytochrome C and a related protein, amidohydrolase family protein, SLC13 family permease, components of the anaerobic DMSO reductase system (DmsC and DmsD), and a molybdopterin-dependent oxidoreductase. The presence of multiple cytochrome C-related proteins suggests that clade 3 may have distinct electron transport capabilities, potentially adapting to specific environmental conditions. The anaerobic DMSO reductase system indicates enhanced anaerobic respiration capabilities, which could provide an advantage in low-oxygen environments.

Furthermore, our analysis revealed likewise a pattern of enzyme and protein gene introgression from *C. lari* into the three clades of *C. coli*, indicating potential functional adaptations and metabolic diversification within the species.

Clades 1 and 2 shared the introgression of a 6-hydroxymethylpterin diphosphokinase MptE-like protein. This enzyme is involved in the folate biosynthesis pathway, specifically in the conversion of 6-hydroxymethylpterin to 6-hydroxymethyldihydropterin pyrophosphate. The acquisition of this enzyme may enhance folate metabolism in these clades.

Clade 2 exhibited unique introgression of a putative membrane protein and a putative reverse transcriptase. While the specific functions of these proteins are not fully elucidated, the presence of a reverse transcriptase suggests potential involvement in genetic mobility or phage defense mechanisms. This could confer increased adaptability or resistance to phage infections in clade 2 isolates.

Several enzyme genes were found to be introgressed into both clades 2 and 3, indicating a potentially significant evolutionary event. These include an ATP-binding protein (AAA domain), which may be involved in various cellular processes, including protein degradation, membrane fusion, and DNA replication. A membrane-bound *O*-acyl transferase (MBOAT family) was also identified, likely playing a role in lipid biosynthesis or modification, potentially altering membrane composition or cell surface properties. The CRISPR/Cas system-associated endoribonuclease Cas2 type II NMENI was another introgressed protein, suggesting enhanced defense capabilities against foreign genetic elements in these clades. Additionally, beta-1,4-*N*-acetylgalactosaminyltransferase (*cgtA*) and glycosyltransferase family 2 enzyme genes were found, which are involved in glycosylation processes. These enzymes may affect cell surface properties, host interactions, and immune evasion strategies.

Clade 3 exhibited unique introgression of several proteins related to bacterial adhesion and secretion systems. These include hemagglutinin domain-containing protein and filamentous hemagglutinin *N*-terminal domain-containing protein, which are typically involved in bacterial adhesion to host cells, suggesting enhanced colonization capabilities in clade 3. Furthermore, two ShlB/FhaC/HecB family hemolysin / secretion activation proteins were identified, which are associated with the two-partner secretion (TPS) system involved in the secretion of large virulence proteins. Their presence indicates potential enhancements in virulence factor secretion and host interaction mechanisms in clade 3.

### Analysis of clade differences in virulence-associated genes

**Fig. 6:**
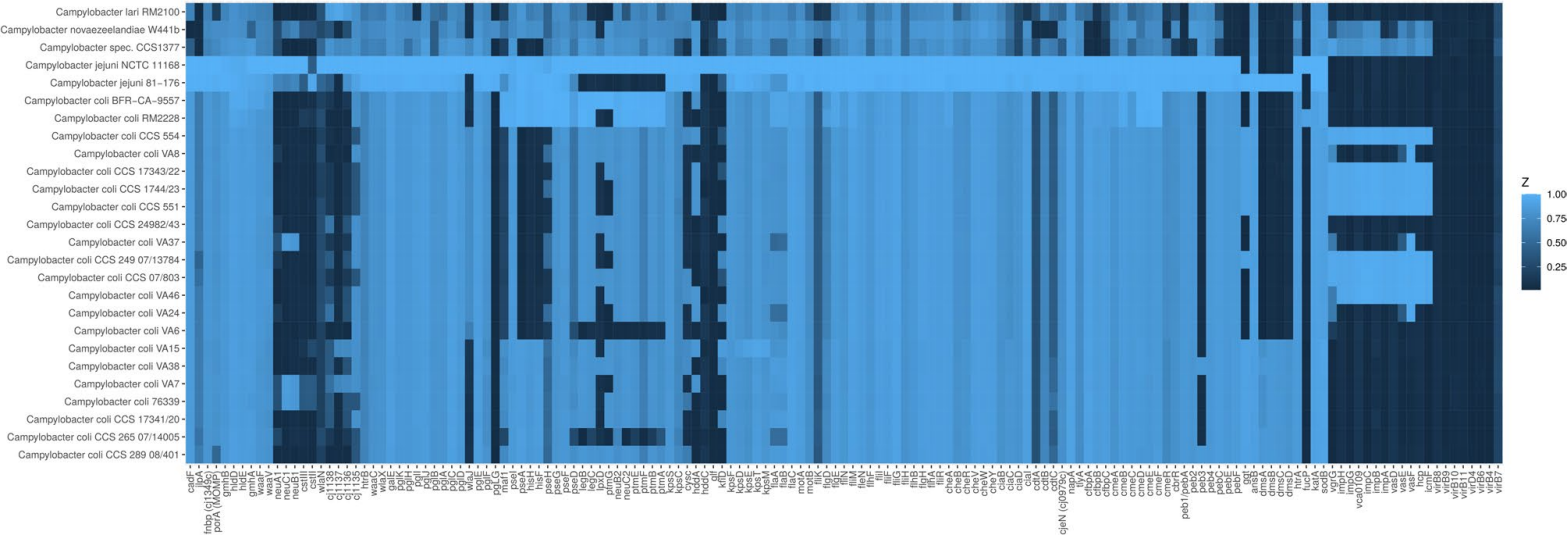
Heatmap indicating the presence of genes encoding established *Campylobacter* virulence associated factors. The *Campylobacter* genomic sequences are organized in phylogenetic order based on the dendrogram presented in Fig. 1. The columns represent 151 genes encoding for virulence-associated factors in a broad sense (legend see Supplementary Table 3). The intensity of the blue color corresponds to the % identity from the BLAST analysis.

Our analysis revealed distinct patterns in the distribution of virulence-associated genes across the three clades of *C. coli* (Fig. 6, Supplementary Table 3). These differences were particularly notable in genes related to lipooligosaccharide (LOS) synthesis, capsule polysaccharide biosynthesis, and various secretion systems, potentially influencing the pathogen’s virulence and host adaptation.

Clades 2 and 3 exhibited general variability in several genes associated with LOS synthesis and sialic acid metabolism. These included putative UDP-*N*-acetylglucosamine 2-epimerase (NeuC1) and sialic acid synthase (NeuB1), which are crucial for sialic acid biosynthesis. Sialic acids play a vital role in bacterial mimicry of host cell surfaces, potentially aiding in immune evasion. Four putative glycosyltransferases (Cj1135, Cj1136, Cj1137c, and Cj1138) involved in LOS synthesis also showed variability. The presence/absence of these enzymes suggests that clades 2 and 3 may have varying abilities to modify their surface structures, potentially affecting host-pathogen interactions.

Additionally, UDP-3-*O*-[3-hydroxymyristoyl] glucosamine *N*-acyltransferase (DegT/LpxD Cj1321) and legionaminic acid biosynthesis protein (PtmG Cj1324) showed variability in these clades. These enzymes are involved in the synthesis of unique surface glycans, which may contribute to antigenic diversity and host colonization efficiency.

Several genes in the capsule polysaccharide biosynthesis locus were predominantly absent across all three clades. These included cytidine diphosphoramidate kinase (Cysc Cj1415c), D-glycero-alpha-D-manno-heptose 7-phosphate kinase (HddA Cj1425c), D-glycero-D-manno-heptose 1-phosphate guanosyltransferase (HddC Cj1423c), UDP-galactopyranose mutase (Glf Cj1439c), and UDP-glucose 6-dehydrogenase (KfiD Cj1441c). The absence of these genes suggests potential alterations in capsule structure across all clades, which could impact immune evasion and environmental persistence.

Clade 2 uniquely possessed the putative integral membrane protein WlaJ (Cj1122c), which may be involved in capsular polysaccharide export. Notably, type VI secretion system components were present in many clade 2 isolates as well as in the isolate CCS1377. The type VI secretion system is known to play a role in bacterial competition and host cell interaction, suggesting that clade 2 isolates may have enhanced competitive abilities in mixed bacterial populations.

Clades 1 and 3 shared the presence of several genes absent in clade 2. These included motility accessory factor (Maf1, Cj1318), which is involved in flagellar assembly and bacterial motility. The presence of genes encoding the pseudaminic acid biosynthesis proteins PseA (Cj1316c) and PseH (*N*-acetyltransferase, Cj1313) suggests the capability to synthesize pseudaminic acid, a sialic-acid-like sugar that plays a role in the glycosylation of flagellin. This glycosylation is crucial for flagellar assembly and function, potentially enhancing motility and host colonization in clades 1 and 3. The presence of imidazole glycerol phosphate synthase subunits (HisH Cj1315c and HisF Cj1314c) suggests intact histidine biosynthesis pathways in the clades 1 and 3, which may provide a metabolic advantage in certain host environments.

The gamma-glutamyltransferase gene (*ggt*) was present in all clade 3 and most clade 2 isolates. Ggt is involved in glutamat and glutathione metabolism. The anaerobic dimethyl sulfoxide reductase gene (*dmsABCD*) was exclusively found in all clade 3 isolates, potentially providing an advantage in anaerobic environments. The gene encoding Peb3, an antigenic surface protein, was mostly absent, being present in only two of the clade 3 isolates as well as *C. jejuni*. This absence might affect the immunogenicity of these strains. Importantly, no type IV secretion system component genes were detected in any of the three clades, and all three clades notably lacked the genes encoding the putative integral membrane protein PglG/WlaM (Cj1119c) and fucose permease (*fucP*), which were present in *C. jejuni*.

### Antimicrobial resistance gene distribution across the *C. coli* clades 2 and 3 and susceptibility testing

**Fig. 7:**
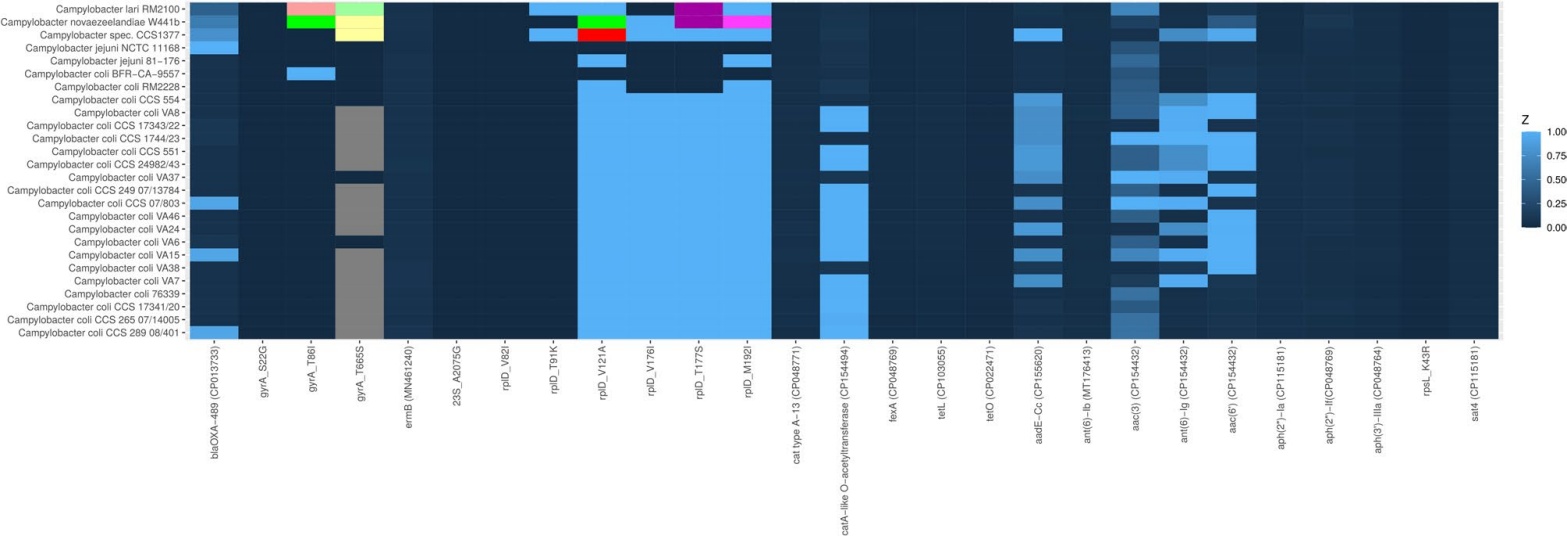
Heatmap indicating the presence of *Camyplobacter* antimicrobial resistance genes. The *Campylobacter* genomic sequences are organized in phylogenetic order based on the dendrogram presented in Fig. 1. The columns represent 20 antimicrobial resistance genes or antimicrobial susceptibility associated point mutations (legend see Supplementary Table 4). The intensity of the blue color corresponds to the % identity from the BLAST analysis with the following exceptions: antique pink - *gyrA* T86V, dark green - *gyrA* T86S, gray - sequence segment absent due to shorter gene, light green - *gyrA* T665L, pale yellow - *gyrA* T665K, red - *rplD* V121M, dark green - *rplD* V121S, violet - *rplD* T177N, magenta - *rplD* M192F

The distribution of antimicrobial resistance (AMR) genes varied significantly among the two clades of *C. coli* (Fig. 7, Supplementary Table 4). Regarding *bla*_OXA_-genes, the beta-lactamase gene *bla*_OXA−489_ was detected by ResFinder in only three genomes of the test population. As indicated by our BLAST-results depicted in heatmap Fig. 7 the presence of other beta-lactamase genes e.g. *bla*_OXA−61_ can also be excluded due to the sequence identity between *bla*_OXA_-genes.

Macrolide resistance genes showed a distinct pattern across the clades. The *ermB* gene (rRNA adenine-*N*-6-methyltransferase) and the 23 S ribosomal RNA A2075G point mutation were absent in all isolates. Similarly, the *rplD* V82I and T91K point mutations were not detected. However, the *rplD* point mutations V121A, V176I, T177S, and M192I were ubiquitous in all clade 2 and 3 isolates.

Regarding chloramphenicol resistance determinants, all isolates were negative for the *fexA* chloramphenicol/florfenicol efflux major facilitator superfamily (MFS) and the Cat type A-13 chloramphenicol-acetyltransferase gene. In contrast, most clade 2 and 3 genomes, with the exception of three isolates, carried a CatA-like *O*-acetyltransferase chloramphenicol-acetyltransferase.

Aminoglycoside resistance genes were predominantly found in clade 2 isolates. The aminoglycoside 6’-*N*-acetyltransferase Aac(6’) and the aminoglycoside nucleotidyltransferase Ant [6]-Ig genes were particularly prevalent in this clade. Additionally, three clade 2 isolates harbored the Aac [3] family *N*-acetyltransferase gene. Clade 2 isolates contained between one and three aminoglycoside-associated resistance genes each, while the tested Slovenian clade 3 isolates lacked these genes. All other tested aminoglycoside resistance-associated genes were negative. Notably, isolate CCS1377 carried both the aminoglycoside 6’-*N*-acetyltransferase Aac(6’) and the aminoglycoside 6-adenylyltransferase AadE-Cc genes, the latter showing high sequence identity to the aminoglycoside nucleotidyltransferase Ant [6]-Ig gene.

Tetracycline resistance genes *tetL* and *tetO* were absent in all tested genomes. Similarly, the streptothricin resistance gene *sat4* (streptothricin *N*-acetyltransferase) and the streptomycin resistance-associated RpsL 30 S ribosomal protein S12 K43R point mutation were not detected in any of the isolates. Notably, none of the isolates harbored a quinolone resistance-associated *gyrA* point mutation.

Based on the established EUCAST and CLSI breakpoints for ciprofloxacin, erythromycin, and tetracycline, it can be definitively stated that, among the tested strains, excluding the reference strains, no resistances were observed (Supplementary Table 5). Regarding the correlation between the presence of antimicrobial resistance (AMR) genes and the observed inhibition zone diameters, a correlation can only be noted between the presence of bla_OXA−489_ in CCS 07/803 and CCS 289 08/401, which exhibited relatively small inhibition zones for penicillin and ampicillin. Otherwise, there is no correlation between the presence of various *rplD* point mutations and macrolide resistance, nor between the presence of the CatA-like *O*-acetyltransferase chloramphenicol-acetyltransferase gene and reduced inhibition zones for chloramphenicol or florfenicol. Additionally, the differing presence of aminoglycoside resistance genes in clade 2 compared to clade 3 does not manifest in phenotypic differences in inhibition zone diameters. It is important to note that ResFinder has limitations, as it does not perform functional predictions. This means that there may be resistance genes with deletions that remain functional but were not reported by the ResFinder pipeline if their similarity falls below the 10% threshold. Conversely, a gene similarity of over 90% results in a reported antimicrobial resistance (AMR) gene, which may not be functional if mutations occur in critical functional domains. This discrepancy may help explain the differences observed between phenotypic resistance and the presence of AMR genes. The cefazolin resistance identified in the API was confirmed through agar diffusion testing.

### Phenotypic characterization

We characterized the available isolates from clades 2 and 3, as well as CCS1377, in comparison with two reference strains from clade 1 of *C. coli* and *C. jejuni*, respectively. This characterization focused on phenotypic traits such as colony morphology, growth dynamics, motility, autoagglutination, biofilm formation, and water survival. Due to the limited number of available isolates within each clade, it is challenging to make definitive qualitative and quantitative statements about phenotypic differences; however, some trends can be observed.

Regarding colony morphology, it was observed that clade 3 isolates exhibited colonies that were generally circular, raised, and opaque, similar to those in clade 1 as well as *C. jejuni* and CCS1377. In contrast, colonies in clade 2 tend to be irregular, flat, and translucent (Supplementary Figure 2).

When quantifying the growth kinetics of the isolates in Mueller Hinton broth, we observed considerable variability in the maximum growth rates among the isolates. Additionally, no statistically significant differences were found between the clades or with CCS1377 (see Supplementary Figure 3). In contrast, the maximum cell mass that was reached during growth kinetics (Supplementary Figure 4) was significantly lower for the clade 3 isolates compared to those of clade 2. Furthermore, clade 3 isolates appears to reach a maximum cell mass that is more comparable to the *C. jejuni* isolates.

A higher motility was observed in all strains at 42 °C than at 37 °C. In six of the tested isolates, a statistically significant difference between motility at 37 °C and 42 °C was observed (p-values < 0.05). Each of the three clade 3 isolates displayed higher motility than any of the clade 1 and clade 2 isolates. CCS1377 and BfR-CA-9557 were non-motile (Supplementary Figure 5).

The autoagglutination capacity of the isolates proved to be highly temperature-dependent (Supplementary Figure 6). Generally, a more pronounced autoagglutination was observed at 37 °C compared to 42 °C. In nine of the tested isolates, a statistically significant difference between autoagglutination ability at 37 °C and at 42 °C was observed (p-values < 0.05). Two of the three clade 3 isolates and the *C. jejuni* isolates exhibited high agglutination rates exceeding 30% at 37 °C, while all clade 2 isolates demonstrated low agglutination rates of less than 5%. Consequently, clade 3 isolates showed a statistically significant higher ability for autoagglutination compared to clade 2 isolates at both temperatures (p-values < 0.05). Additionally, clade 3 isolates displayed similarly high autoagglutination abilities as the two *C. jejuni* reference strains. CCS1377 exhibits a relatively weak autoagglutination ability, similar to the clade 2 isolates.

The ability to form biofilm among the tested isolates showed considerable variability, and no statistically significant differences were observed between the clades (see Supplementary Figure 7). While one isolate from clade 2 (CCS1744/23) exhibited stronger biofilm formation, while the overall levels of biofilm formation in clade 2 isolates were comparable to those from clade 3.

In the assessment of water survival at 4 °C, the two *C. jejuni* reference strains dominate with water survival abilities at day 2 of up to 80% and 4.6% (Supplementary Figure 8 A). The rest of the tested isolates displayed water survival abilities of 3.0% and below at day 2. When the *C. jejuni* strains are excluded from the graphical representation (Supplementary Figure 8B), it becomes clear that the clade 3 isolates exhibit a significantly higher water survival ability at 4 °C compared to the clade 1 and 2 isolates. Isolate CCS1377 displayed similar water survival ability as clade 1 and 2 *C. coli* isolates.

The Analytical Profile Index (API Campy) results revealed distinct biochemical profiles among the clades 2 and 3 of *C. coli*. Gamma-glutamyltransferase (Ggt) activity was a prominent feature, with all isolates testing positive except for three from clade 2. This biochemical observation aligned with the genomic data, which confirmed the presence of the *ggt* gene in most isolates. Notably, isolate CCS554 harbored a disrupted *ggt* gene, potentially explaining its negative Ggt activity despite the gene’s presence.

Proprionate assimilation capabilities varied among the clades. Five out of seven clade 2 isolates demonstrated the ability to assimilate proprionate, whereas this trait was absent in all clade 3 isolates and the other reference strains.

Citrate assimilation also showed a different distribution pattern across the clades. All three clade 3 isolates and four out of seven clade 2 isolates exhibited the ability to assimilate citrate. This observation indicates a more widespread citrate utilization capability in clade 3 compared to clade 2, potentially reflecting differences in their ecological niches or evolutionary histories.

The API Campy system successfully identified all clade 2 and 3 isolates as *C. coli*, confirming the accuracy of the initial species classification. However, isolate CCS1377 presented an intriguing exception. Due to its negative Gamma-glutamyltransferase (Ggt) reaction, inability to assimilate succinate, and susceptibility to cefazoline, the API system identified CCS1377 as *C. lari* with a high confidence level of 99.7%.

## Discussion

In the study presented here, we phylogenetically classified ten of eleven Slovenian *Campylobacter* isolates from environmental waters as *C. coli* of Clades 2 and 3, while one isolate, CCS1377, might represent a new *Campylobacter* species. We examined these isolates under three key aspects: gene introgression from other *Campylobacter* species, the presence of well-characterized virulence-associated factors and antimicrobial resistance genes, as well as fundamental phenotypic characteristics.

In addition to the previously described admixture of *C. jejuni* genes [1, 2, 5], which ranged in our study from 6.58 to 9.46% for clade 2 isolates and from 5.39 to 6.66% for clade 3 genomes (as shown in Table 2; Fig. 3B and C), we also identified an admixture of *C. lari* genes in the clade 2 genomes, ranging from 0.44 to 1.59%, and in the clade 3 genomes, ranging from 0.31 to 1.39%. A BLAST analysis of the clade-specific genes with apparent *C. lari* origin revealed that most hits were found in the genomes of *C. lari* NCTC 11,845, *C. lari* RM16712, and *C. lari* subsp. *concheus* RM19247. Notably, hits were also identified in the genome of isolate CCS1377, which might belong to a further new *Campylobacter* species. Since our approach to admixture analysis heavily relies on the genomes stored in the GenBank database, it is possible that the introgression may have occurred not only from *C. lari* but also (or even instead) from other *Campylobacter* species such as those that might be represented by CCS1377 into the *C. coli* isolates from clades 2 and 3. This possibility warrants further investigation for greater accuracy.

The analysis of virulence factors revealed that for example the gamma-glutamyltransferase gene (*ggt*) was present in all clade 3 isolates and most clade 2 isolates, which was confirmed by our API results. Additionally, the anaerobic dimethyl sulfoxide reductase genes (*dmsABCD*) were exclusively found in all clade 3 isolates. Our 1,000-mer-based admixture analysis indicated that these genes were the result of introgression from *C. jejuni*. Furthermore, there was notable variability between clades 2 and 3 in the putative UDP-*N*-acetylglucosamine 2-epimerase (NeuC1), sialic acid synthase (NeuB1), and the putative glycosyltransferases (Cj1135, Cj1136, Cj1137c, and Cj1138), which are associated with lipooligosaccharide (LOS) synthesis and sialic acid metabolism. These findings are consistent with the results reported by Skarp-de Haan and colleagues. They conducted a comparative genomic analysis of the first fully closed *C. coli* clade 3 genome from isolate 76,339. Their findings revealed several specific genomic regions, including genes involved in various metabolic functions such as the gamma-glutamyltranspeptidase (*ggt*) gene, biotin sulfide reductase (*bsr*), cytochrome C (*cytC*), DMSO reductase (*dmsABCD*), two type I restriction modification systems, an active sialyltransferase in the lipooligosaccharide locus, and two serine proteases [33].

In contrast to the genomic analysis of clade 2 and 3 *C. coli* isolates, research on phenotypic characterization remains limited. A small study involving only five clade 2 and three clade 3 isolates from Swedish water plants examined their colony morphology, water survival, biofilm formation, motility, and ability to utilize tricarballylate. Since this study was based on only a few isolates from a single geographical region, Sweden, it would be beneficial to expand the empirical basis, which we have achieved with our study on Slovenian isolates. We were likewise able to demonstrate that clade 2 isolates exhibit irregular, flat, and translucent colonies, whereas clade 3 isolates display colonies that are generally circular, raised, and opaque. These clade differences in colony morphology could be attributed to the presence of the enzymes PseA, PseC, and PseH in the clade 2 isolates, which are involved in protein glycosylation and may influence cell surface properties.

Additionally, the water survival and swarming motility of clade 2 isolates were lower compared to those of clade 3 isolates. Surprisingly, the water survival of the aquatic *C. coli* isolates was lower than that of the two *C. jejuni* reference isolates. This suggests that the habitat of these isolates may not be the water itself, but rather habitats such as protozoa, waterfowl, or other aquatic hosts. This could explain the reduced water survival compared to *C. jejuni*. Further studies are needed to verify this hypothesis. Moreover, we found no significant differences in biofilm formation. Although we did not phenotypically test the ability to utilize tricarballylate, we could also observe in our test cohort that the gene cluster responsible for this, *tcuRCAB*, is present in clade 2 isolates and absent in clade 3 isolates [11].

Furthermore, we expanded the phenotypic parameters in the description of clade differences assessing antimicrobial susceptibility, growth analyses, and autoagglutination. In contrast to the antimicrobial susceptibility testing, where no clade differences were observed, the growth analysis revealed a higher maximum optical density (biomass) in clade 2 isolates compared to clade 3 isolates. Conversely, clade 3 isolates exhibited higher autoagglutination than clade 2. We demonstrated the introgression of genes encoding adhesion and secretion-related proteins in the genomes of clade 3, including hemagglutinin domain-containing proteins and filamentous hemagglutinin *N*-terminal domain-containing proteins. These proteins may be responsible for the observed phenotypical differences in autoagglutination. These factors which are typically involved in bacterial adhesion to host cells, suggest enhanced capabilities of clade 3 isolates to colonize and infect hosts [31]. The eight Swedish isolates have been analyzed for their effects on human HT-29 colon cancer cells by Johansson and colleagues [34]. They reported that clade 2 and 3 isolates exhibited stronger adherence to the colon cancer cells, while only clade 3 isolates induced rapid cell death in vitro. This phenomenon may correlate with the phenotypic differences in autoagglutination and could be traced back to the introgressed genes encoding these adhesion and secretion-related protein-genes. This suggests that clade 3 may exhibit heightened virulence, potentially in host species other than humans, compared to the other two *C. coli* clades. In contrast, no differences were observed among the clades in terms of interleukin-8 induction [34].

It is also important to note that the genes introgressed into *C. coli* isolates of clades 2 and 3 from *C. jejuni* differ from those found in the previously described *C. coli*/*C. jejuni* hybrid strains. These genes include a fused type II restriction-modification system, the fucose permease gene *fucP*, and the multidrug efflux RND transporter permease subunit genes *cmeB*, *cmeE*, and *cmeF* [5].

These findings underscore the role of horizontal gene transfer from *C. jejuni*, *C. lari*, and other *Campylobacter* species such as those that might be represented by CCS1377, highlighting their contribution to the genetic basis of clade-specific traits and their potential impact on pathogenicity and suggesting potential adaptations to different ecological niches.

Future research should focus on elucidating the specific mechanisms driving introgression in agricultural settings, investigating the functional consequences of clade-specific genetic elements, and exploring the potential for targeted interventions based on clade-specific virulence factors. Additionally, further studies on the epidemiology and niche/host range of clade 2 and 3 isolates as well as CCS1377-like isolates could provide valuable information for public health strategies and risk assessment in the context of foodborne illness prevention.

## Electronic supplementary material

Below is the link to the electronic supplementary material.

**Supplementary Material 1**: 31_plotHyb.R R script for analyzing introgression through k-mer analysis.

**Supplementary Material 2**: Clade analysis.r R script for conducting statistical analysis using a linear mixed-effects model.

**Supplementary Material 3**: MLST-based phylogenetic tree of C. coli and C. jejuni isolates. The MLST-based dendrogram includes sequences from the seven clade 2 *C. coli* isolates, three clade 3 isolates, and CCS1377 included in this study. For additional reference, it also features 19 clade 1 isolates, four *C. jejuni* isolates, and three *C. coli*/*C. jejuni* hybrid isolates [35]. In this analysis, *Campylobacter* sp. CCS1377 was found to be closely grouped with *C. coli* clade 2.

**Supplementary Material 4**: Colony morphology. Assessment of colony morphology after 24 h at 37 °C on COS agar was conducted based on the eight criteria: size, shape, edge, elevation, surface, color, opacity, and texture, as established by Sousa and colleagues

**Supplementary Material 5**: Maximum growth rate. The growth analysis using the Cell Growth Quantifier from Aquila Biolabs was conducted for 48 h at 37 °C in MH Broth, with shaking at 150 rpm. The Maximum growth rate, was estimated by measuring the steepest increase in the growth curve. The average maximum growth rates were 0.38 ± 0.09(SD) OD/h for clade 1, 0.39 ± 0.10(SD) OD/h for clade 2 and 0.41 ± 0.07(SD) OD/h for clade 3. No statistical significant differences were observed in terms of growth rate among the clades. Growth rate was calculated as follows: $\:\mu\:=\frac{ln\frac{{X}_{{t}_{2}}}{{X}_{{t}_{1}}}}{({t}_{2}-{t}_{1})}$

**Supplementary Material 6**: Growth analysis - maximum optical density (OD_**600**_**)**. At 37 °C, the clade 2 isolates demonstrate, on average, a stronger biomass production compared to the clade 3 *C. coli* isolates (p-value < 0.001). The average maximum OD values were 2.2 ± 0.16(SD) OD_600_ for clade 1, 2.41 ± 0.15(SD) OD_600_ for clade 2 and 1.97 ± 0.08(SD)OD_600_ for clade 3. Clade 2 strains reached a statistically significant higher OD_600_ than clade 3 strains and *C. jejuni* strains with p-values less than 0.01. A p-value of less than 0.01 is denoted by “**”.

**Supplementary Material 7**: Swarming motility at 37 °C and 42 °C. The swarming motility of the test strains demonstrated a significant dependence on temperature. All strains exhibited higher motility at 42 °C compared to 37 °C, with several strains showing statistically significant increases in motility at 42 °C. Specifically, the following strains had significant differences: CCS24982/43 (*p* < 0.05) and CCS24907/13784 (*p* < 0.05). Clade 3 *C. coli* isolates exhibited significantly higher motility at 37 °C compared to clade 2 isolates (*p* < 0.001). Notably, *Campylobacter* sp. CCS1377 and *C. coli* BfR-CA-9557 were found to be non-motile. The average swarming motility diameters at 42 °C were 11.9 ± 11.9(SD) mm for clade 1, 22.4 ± 2.7(SD) mm for clade 2, and 33.2 ± 1.6(SD) mm for clade 3. At 37 °C, the average swarming motility diameters were 9.7 ± 9.7(SD) mm for clade 1, 15.4 ± 1.7(SD) mm for clade 2, and 28.2 ± 2.3 (SD) mm for clade 3. A p-value of less than 0.05 is denoted by “*”, and a p-value of less than 0.001 by “***”.

**Supplementary Material 8**: Relative autoagglutination of the tested campyobacter isolates. Autoagglutination is highly sensitive to temperature, being significantly more pronounced at 37 °C compared to 42 °C. All strains demonstrated stronger autoagglutination at 37 °C, with several strains showing statistically significant increases. These include CCS1377 (*p* < 0.0001), 81–176 (*p* < 0.05), RM2228 (*p* < 0,05), CCS554 (*p* < 0.01), CCS17343/22 (*p* < 0.01), CCS24907/13784 (*p* < 0.01), CCS07/803 (*p* < 0.05), CCS26507/14005 (*p* < 0.01) and CCS28908/401 (*p* < 0.001). A p-value of less than 0.05 is denoted by “*”, a p-value of less than 0.01 by “**”, and a p-value of less than 0.001 by “***”. The autoagglutination is particularly strong in *C. jejuni* and one of the clade 1 *C. coli* isolates (BfR-CA-9557). Additionally, isolates from clade 3 exhibit significantly stronger autoagglutination on average than those from clade 2 (*p* < 0.05). The average relative autoagglutination values at 42 °C were 17.2 ± 15(SD) for clade 1, 2.5 ± 0.2 (SD) for clade 2, and 5.2 ± 3.2 (SD) for clade 3. At 37 °C, the average relative autoagglutination values were 38.1 ± 32(SD) for clade 1, 3.8 ± 0.4(SD) for clade 2, and 24.3 ± 14.4 (SD) for clade 3.

**Supplementary Material 9**: Biofilm formation of the Slovenian *** Campylobacter*isolates**.To assess biofilm formation, bacteria were incubated in Mueller-Hinton broth for 48 h at 37 °C under microaerophilic conditions. Biofilms were subsequently stained with crystal violet. The experiments were conducted in technical quadruplets and biological triplicates. The bars represent the means ± standard deviations of three biological replicate experiments. The differences in biofilm formation among the isolates exhibited considerable variability, and comparisons between the different clades did not yield statistically significant differences. The average absorbance values were 0.65 ± 0.21(SD) for clade 1, 0.51 ± 0.21 (SD) for clade 2, and 0.47 ± 0.13 (SD) for clade 3.

**Supplementary Material 10**: Water survival ability of the tested Slovenian Campylobacter isolates. The diagrams illustrate the percentage of colony forming units [cfu] in relation to the cfu count recorded immediately after plating on day 0. For comparison, two clade 1 *C. coli* isolates and two *C. jejuni* reference isolates were included in the analysis. Figure 8 A shows that the *C. jejuni* reference strains exhibit significantly better survival in water at 4 °C. For clarity, these *C. jejuni* reference isolates have been omitted in Fig. 8B. The clade 3 isolates exhibit superior water survival compared to isolates from clade 1 and *Campylobacter* CCS1377, as well as significantly better water survival than the clade 2 isolates (*p* < 0.001). The average water survival rates at day 2 are 0.049 ± 0.033 (SD) % for clade 1, 0.009 ± 0.007(SD) % for clade 2, and 1.322 ± 0.615(SD) % for clade 3. The average water survival rates at day 4 are 0.001 ± 0.001(SD) % for clade 1, 0.0001 ± 0.0003(SD) % for clade 2 and 0.013 ± 0.008(SD) % for clade 3. A p-value of less than 0.05 is denoted by “*”, and a p-value of less than 0.001 by “***”.

**Supplementary Material 11**: Supplementary Tables 1 - 5

## Data Availability

Sequence data supporting the findings of this study have been deposited in NCBI GenBank under the Bioproject Accession PRJNA1105365. The Campylobacter isolates characterized in this study are deposited at the Leibniz Institute DSMZ-German Collection of Microorganisms and Cell Cultures GmbH.
