# Supplementary figures and images for: Characterization of aquatic clade 2 and 3 Campylobacter coli isolates from Slovenia reveals admixture with other Campylobacter species
Source: BMC Microbiol. 2025 May 24;25:322. doi: 10.1186/s12866-025-04042-z (PMC12102941; doi:10.1186/s12866-025-04042-z)

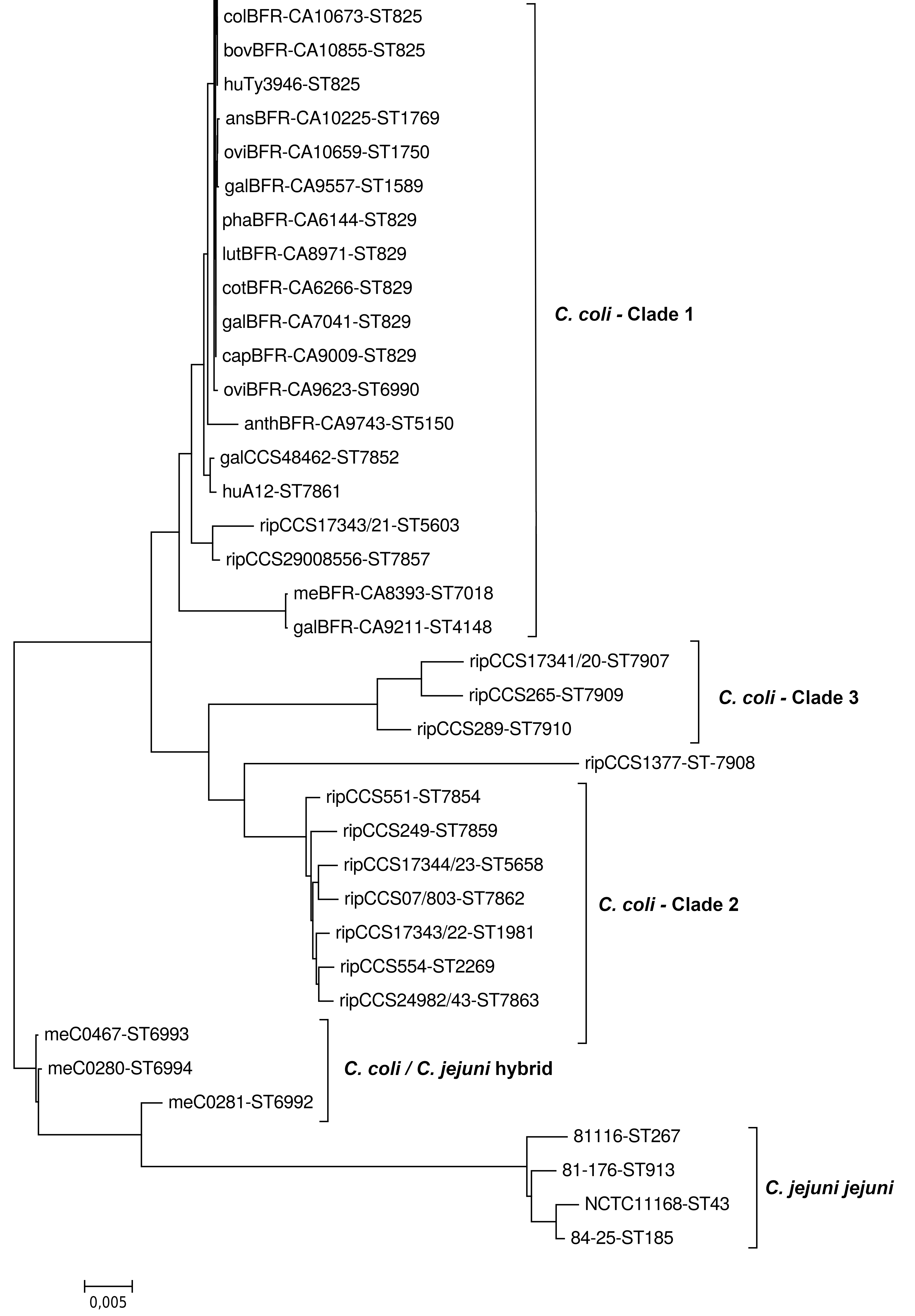

Supplement: Supplementary file 3 — Supplementary Material 3: MLST-based phylogenetic tree of C. coli and C. jejuni isolates. The MLST-based dendrogram includes sequences from the seven clade 2 C. coli isolates, three clade 3 isolates, and CCS1377 included in this study. For additional reference, it also features 19 clade 1 isolates, four C. jejuni isolates, and three C. coli/C. jejuni hybrid isolates [35]. In this analysis, Campylobacter sp. CCS1377 was found to be closely grouped with C. coli clade 2. [file 12866_2025_4042_MOESM3_ESM.jpg]

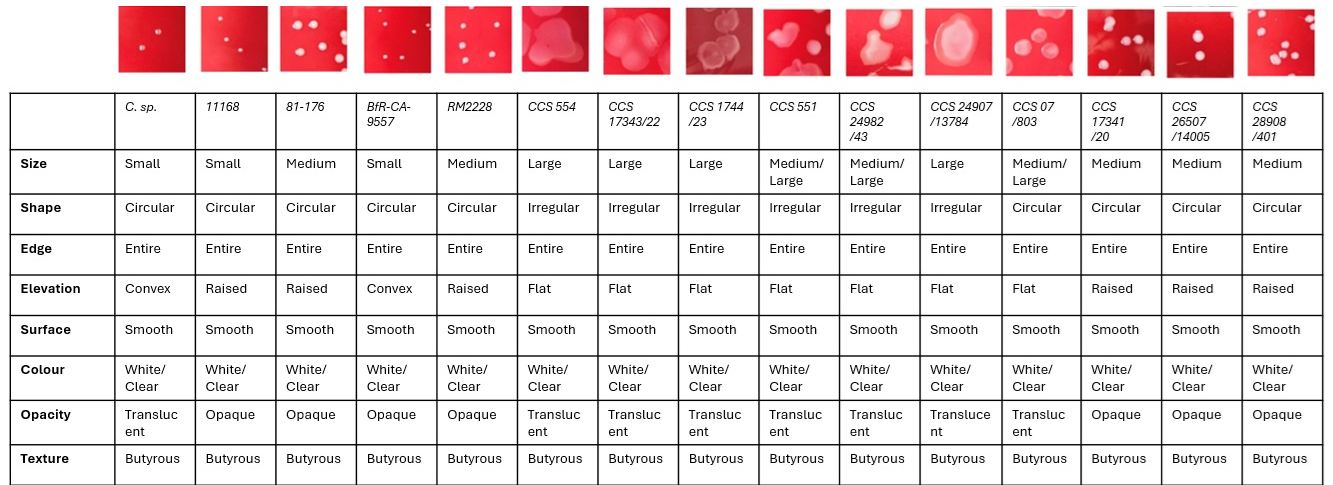

Supplement: Supplementary file 4 — Supplementary Material 4: Colony morphology. Assessment of colony morphology after 24 h at 37 °C on COS agar was conducted based on the eight criteria: size, shape, edge, elevation, surface, color, opacity, and texture, as established by Sousa and colleagues [file 12866_2025_4042_MOESM4_ESM.jpg]

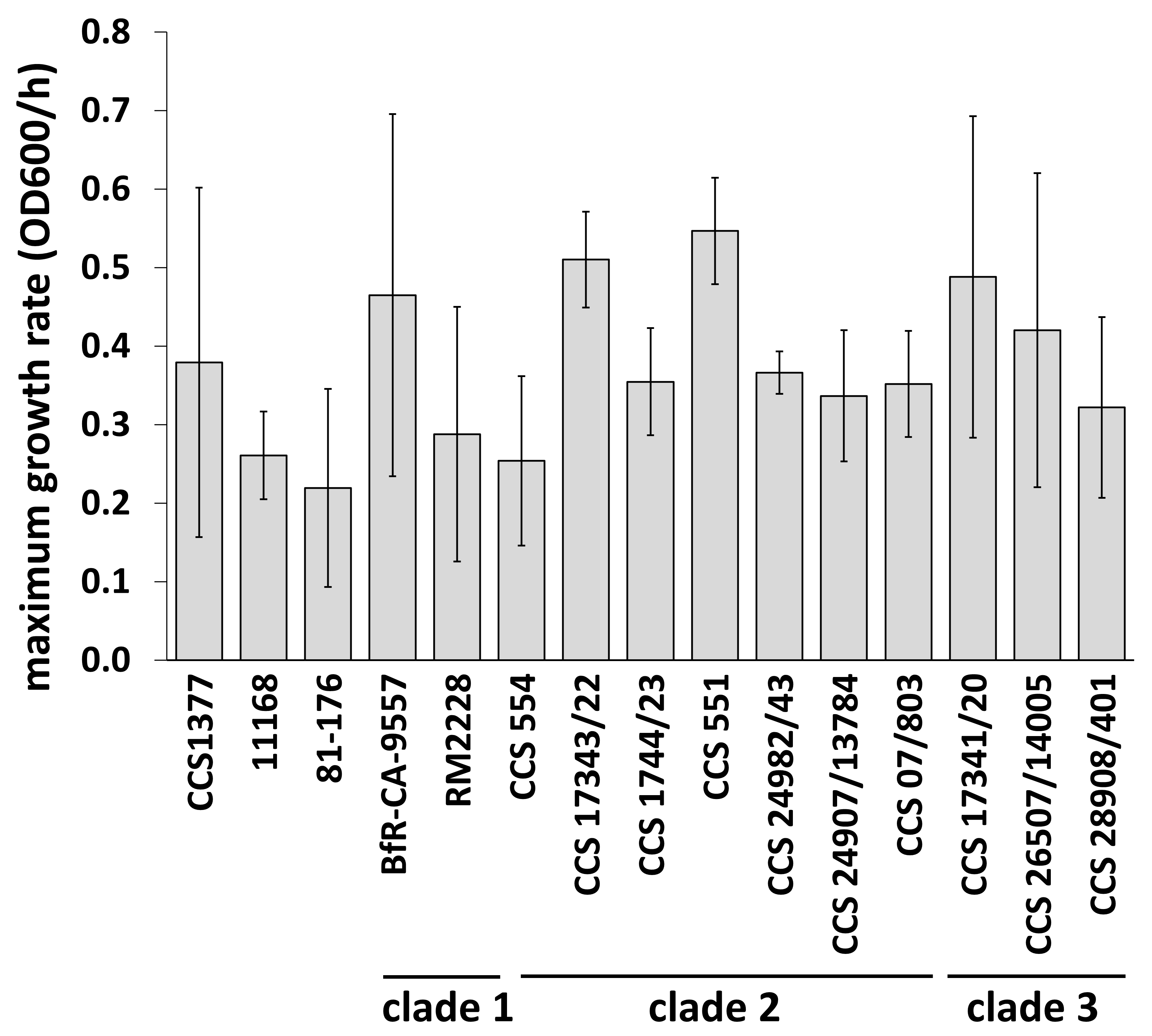

Supplement: Supplementary file 5 — Supplementary Material 5: Maximum growth rate. The growth analysis using the Cell Growth Quantifier from Aquila Biolabs was conducted for 48 h at 37 °C in MH Broth, with shaking at 150 rpm. The Maximum growth rate, was estimated by measuring the steepest increase in the growth curve. The average maximum growth rates were 0.38 ± 0.09(SD) OD/h for clade 1, 0.39 ± 0.10(SD) OD/h for clade 2 and 0.41 ± 0.07(SD) OD/h for clade 3. No statistical significant differences were observed in terms of growth rate among the clades. Growth rate was calculated as follows: $\:\mu\:=\frac{ln\frac{{X}_{{t}_{2}}}{{X}_{{t}_{1}}}}{({t}_{2}-{t}_{1})}$ [file 12866_2025_4042_MOESM5_ESM.jpg]

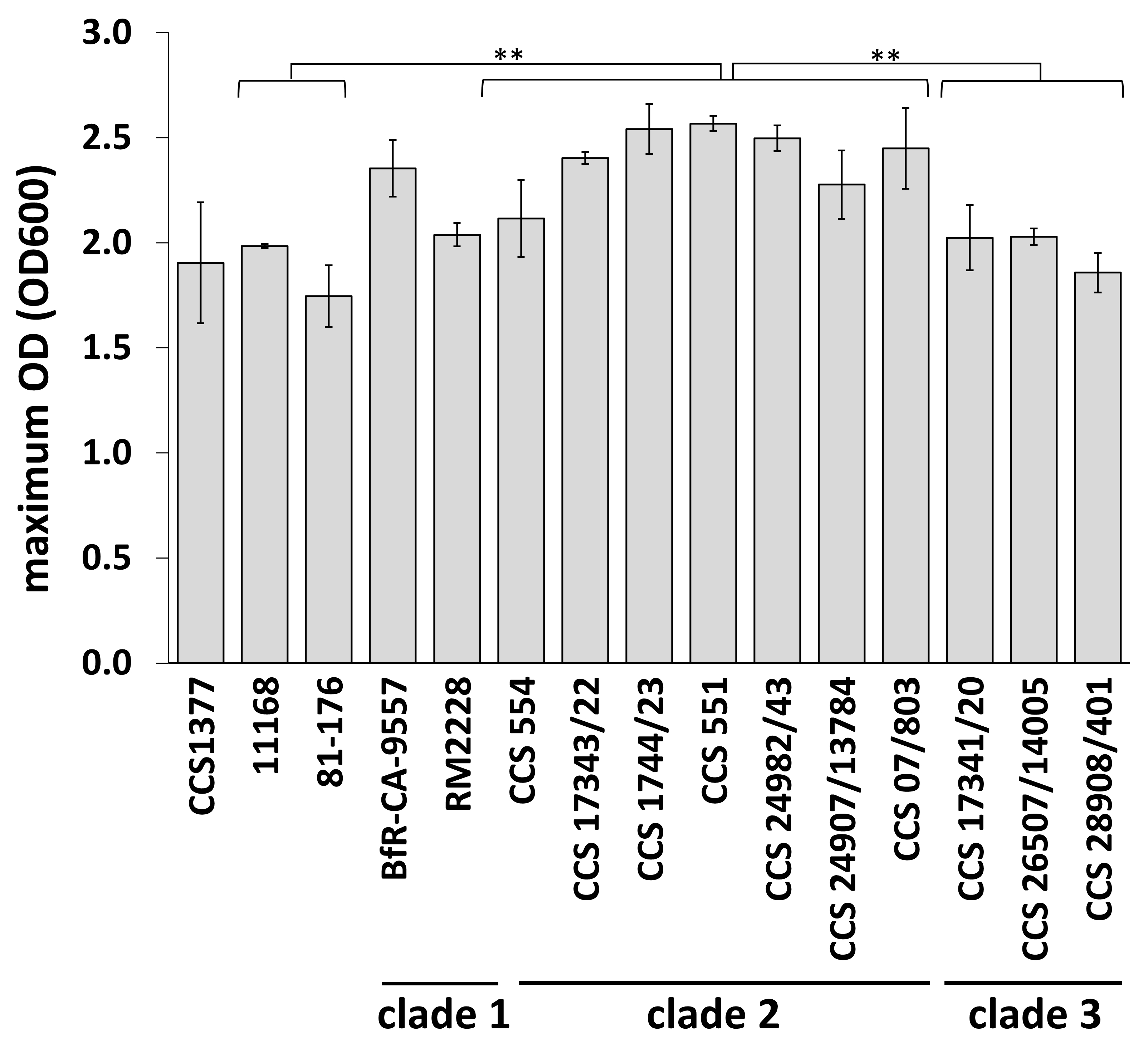

Supplement: Supplementary file 6 — Supplementary Material 6: Growth analysis - maximum optical density (OD600). At 37 °C, the clade 2 isolates demonstrate, on average, a stronger biomass production compared to the clade 3 C. coli isolates (p-value < 0.001). The average maximum OD values were 2.2 ± 0.16(SD) OD600 for clade 1, 2.41 ± 0.15(SD) OD600 for clade 2 and 1.97 ± 0.08(SD)OD600 for clade 3. Clade 2 strains reached a statistically significant higher OD600 than clade 3 strains and C. jejuni strains with p-values less than 0.01. A p-value of less than 0.01 is denoted by “**”. [file 12866_2025_4042_MOESM6_ESM.jpg]

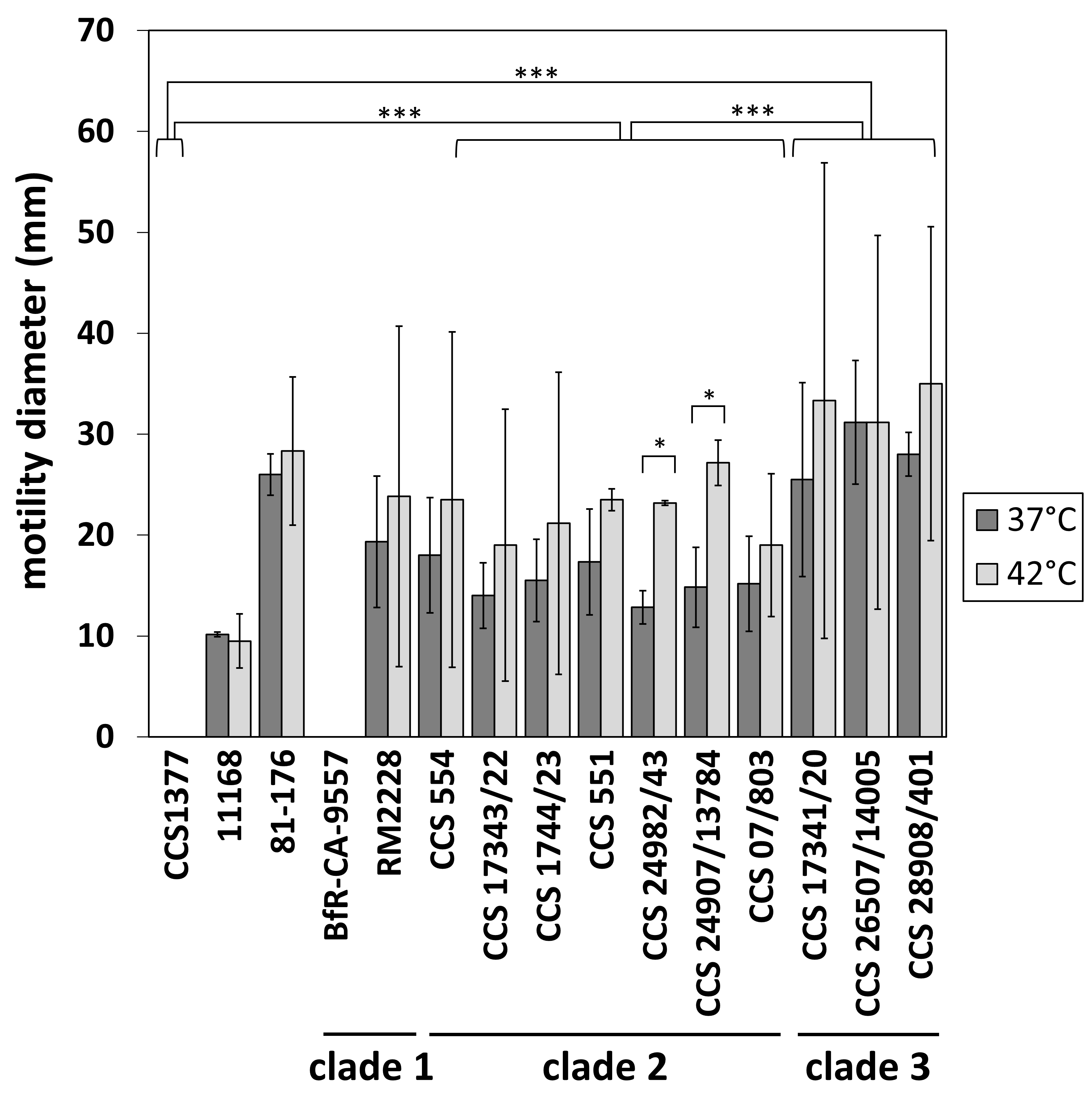

Supplement: Supplementary file 7 — Supplementary Material 7: Swarming motility at 37 °C and 42 °C. The swarming motility of the test strains demonstrated a significant dependence on temperature. All strains exhibited higher motility at 42 °C compared to 37 °C, with several strains showing statistically significant increases in motility at 42 °C. Specifically, the following strains had significant differences: CCS24982/43 (p < 0.05) and CCS24907/13784 (p < 0.05). Clade 3 C. coli isolates exhibited significantly higher motility at 37 °C compared to clade 2 isolates (p < 0.001). Notably, Campylobacter sp. CCS1377 and C. coli BfR-CA-9557 were found to be non-motile. The average swarming motility diameters at 42 °C were 11.9 ± 11.9(SD) mm for clade 1, 22.4 ± 2.7(SD) mm for clade 2, and 33.2 ± 1.6(SD) mm for clade 3. At 37 °C, the average swarming motility diameters were 9.7 ± 9.7(SD) mm for clade 1, 15.4 ± 1.7(SD) mm for clade 2, and 28.2 ± 2.3 (SD) mm for clade 3. A p-value of less than 0.05 is denoted by “*”, and a p-value of less than 0.001 by “***”. [file 12866_2025_4042_MOESM7_ESM.jpg]

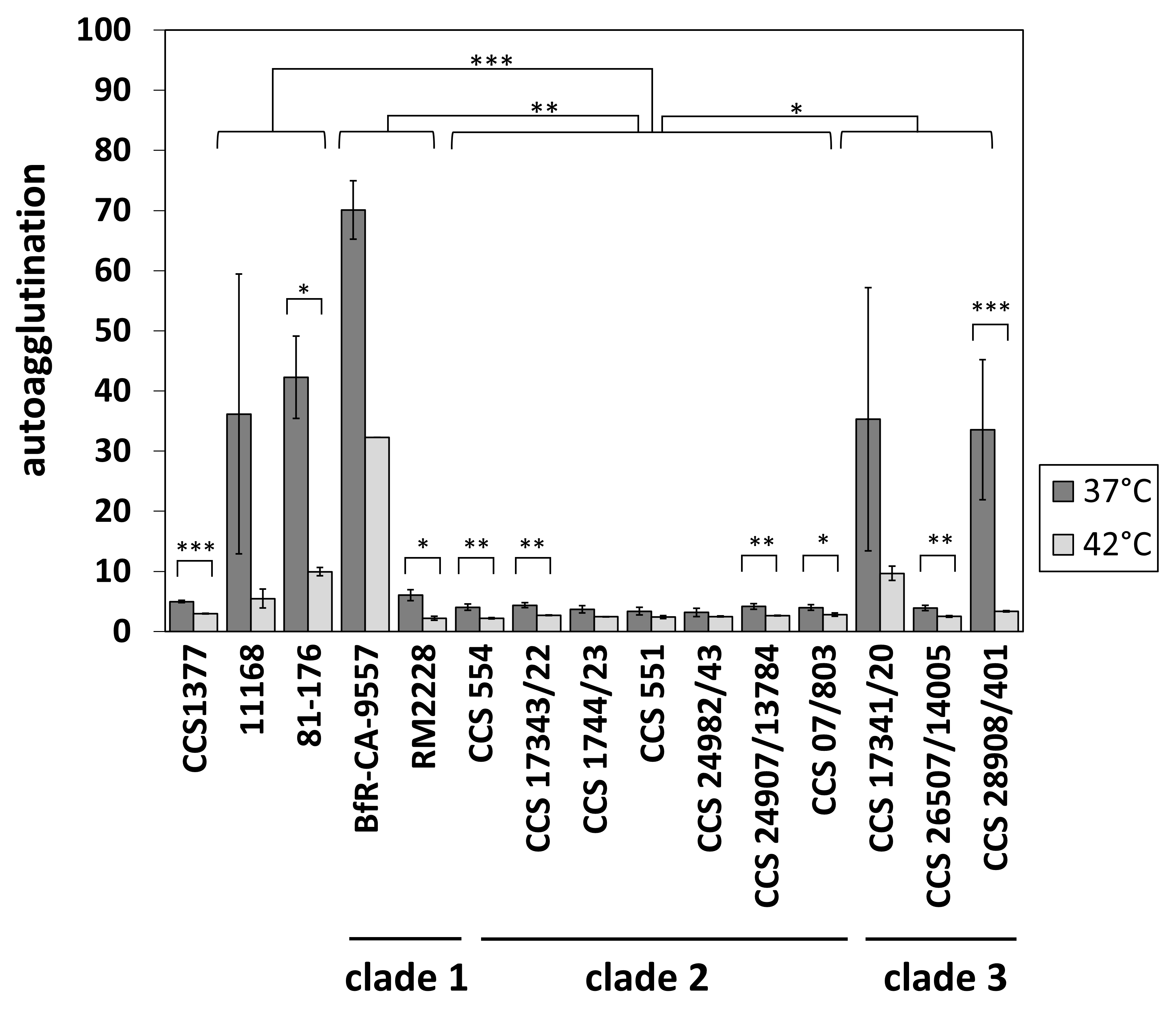

Supplement: Supplementary file 8 — Supplementary Material 8: Relative autoagglutination of the tested campyobacter isolates. Autoagglutination is highly sensitive to temperature, being significantly more pronounced at 37 °C compared to 42 °C. All strains demonstrated stronger autoagglutination at 37 °C, with several strains showing statistically significant increases. These include CCS1377 (p < 0.0001), 81–176 (p < 0.05), RM2228 (p < 0,05), CCS554 (p < 0.01), CCS17343/22 (p < 0.01), CCS24907/13784 (p < 0.01), CCS07/803 (p < 0.05), CCS26507/14005 (p < 0.01) and CCS28908/401 (p < 0.001). A p-value of less than 0.05 is denoted by “*”, a p-value of less than 0.01 by “**”, and a p-value of less than 0.001 by “***”. The autoagglutination is particularly strong in C. jejuni and one of the clade 1 C. coli isolates (BfR-CA-9557). Additionally, isolates from clade 3 exhibit significantly stronger autoagglutination on average than those from clade 2 (p < 0.05). The average relative autoagglutination values at 42 °C were 17.2 ± 15(SD) for clade 1, 2.5 ± 0.2 (SD) for clade 2, and 5.2 ± 3.2 (SD) for clade 3. At 37 °C, the average relative autoagglutination values were 38.1 ± 32(SD) for clade 1, 3.8 ± 0.4(SD) for clade 2, and 24.3 ± 14.4 (SD) for clade 3. [file 12866_2025_4042_MOESM8_ESM.jpg]

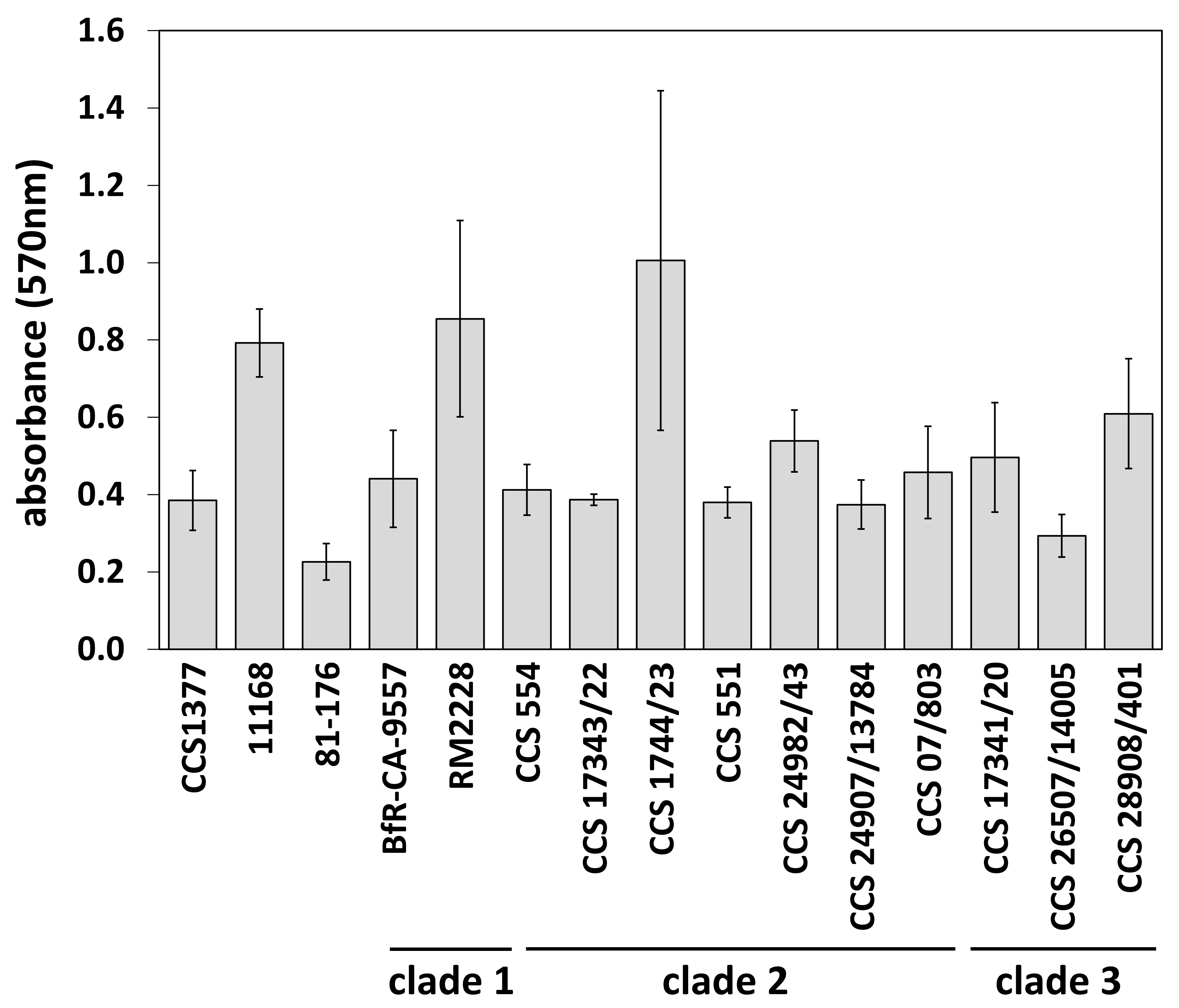

Supplement: Supplementary file 9 — Supplementary Material 9: Biofilm formation of the Slovenian Campylobacterisolates.To assess biofilm formation, bacteria were incubated in Mueller-Hinton broth for 48 h at 37 °C under microaerophilic conditions. Biofilms were subsequently stained with crystal violet. The experiments were conducted in technical quadruplets and biological triplicates. The bars represent the means ± standard deviations of three biological replicate experiments. The differences in biofilm formation among the isolates exhibited considerable variability, and comparisons between the different clades did not yield statistically significant differences. The average absorbance values were 0.65 ± 0.21(SD) for clade 1, 0.51 ± 0.21 (SD) for clade 2, and 0.47 ± 0.13 (SD) for clade 3. [file 12866_2025_4042_MOESM9_ESM.jpg]

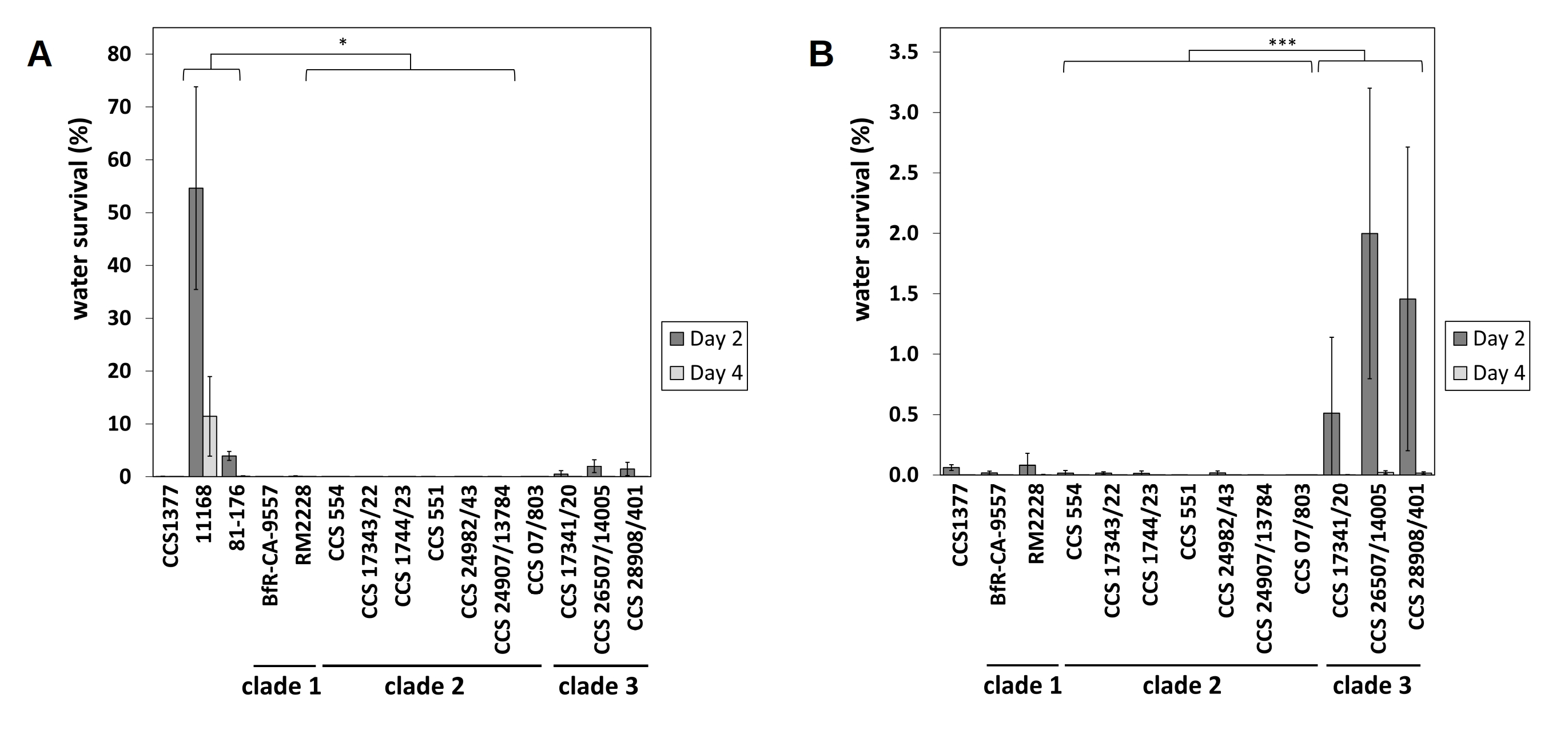

Supplement: Supplementary file 10 — Supplementary Material 10: Water survival ability of the tested Slovenian Campylobacter isolates. The diagrams illustrate the percentage of colony forming units [cfu] in relation to the cfu count recorded immediately after plating on day 0. For comparison, two clade 1 C. coli isolates and two C. jejuni reference isolates were included in the analysis. Figure 8 A shows that the C. jejuni reference strains exhibit significantly better survival in water at 4 °C. For clarity, these C. jejuni reference isolates have been omitted in Fig. 8B. The clade 3 isolates exhibit superior water survival compared to isolates from clade 1 and Campylobacter CCS1377, as well as significantly better water survival than the clade 2 isolates (p < 0.001). The average water survival rates at day 2 are 0.049 ± 0.033 (SD) % for clade 1, 0.009 ± 0.007(SD) % for clade 2, and 1.322 ± 0.615(SD) % for clade 3. The average water survival rates at day 4 are 0.001 ± 0.001(SD) % for clade 1, 0.0001 ± 0.0003(SD) % for clade 2 and 0.013 ± 0.008(SD) % for clade 3. A p-value of less than 0.05 is denoted by “*”, and a p-value of less than 0.001 by “***”. [file 12866_2025_4042_MOESM10_ESM.jpg]
