# Supplementary material for: Characterization of aquatic clade 2 and 3 Campylobacter coli isolates from Slovenia reveals admixture with other Campylobacter species
Source: BMC Microbiol. 2025 May 24;25:322. doi: 10.1186/s12866-025-04042-z (PMC12102941; doi:10.1186/s12866-025-04042-z)
Supplement: Supplementary file 11 — Supplementary Material 11: Supplementary Tables 1 - 5 [file 12866_2025_4042_MOESM11_ESM.docx]

**Supplementary Table 1. *C. jejuni* genes**

| **locus tag** | **GenBank accession** | **strain** | **putative protein function** | **clades** |
| --- | --- | --- | --- | --- |
| FBF06_04555 | CP040610 | CFSAN093227 | hypothetical protein | 1, 2, *C. jejuni* |
| G3T50_03995 | CP048771 | ZS007 | hypothetical protein | 1, 2, *C. jejuni* |
| NCTC11951_01487 | LR134359 | NCTC11951 | D-lactate dehydrogenase Fe-S protein FAD/FMN-containing protein | 1, 2, *C. jejuni* |
| THJ114_17080 | AP026015 | THJ114 | hypothetical protein (plasmid pTHJ114_1) | 1, 2, *C. jejuni* |
| THJ114_17090 | AP026015 | THJ114 | peptidase C39 (plasmid pTHJ114_1) | 1, 2, *C. jejuni* |
| B11447_13870 | AP028392 | BCH-11447 | hypothetical protein | 1 ,2 |
| HCQ89_08675 | CP050683 | BP18682 | YeeE/YedE family protein | 1, 2 |
| HPY19_08110 | CP053854 | 129108 | hypothetical protein | 1, 2, CCS1377 |
| NCTC13268_01052 | LR134497 | NCTC13268 | methyltransferase small | 1, 2, CCS1377 |
| B11447_13880 | AP028392 | BCH-11447 | hypothetical protein | 2 |
| BLD34_04875 | CP017863 | IF1100 | hypothetical protein | 2, CCS1377 |
| BLD34_04880 | CP017863 | IF1100 | hypothetical protein | 2, CCS1377 |
| CJ14980A_0939 | CP017029 | 14980A | hypothetical protein | 2, CCS1377 |
| CJ14980A_0940 | CP017029 | 14980A | hypothetical protein | 2, CCS1377 |
| E5V15_02940 | CP038862 | SCJK2 | hypothetical protein | 2, CCS1377 |
| G3T50_09060 | CP048772 | ZS007 | hypothetical protein (plasmid pCJ145K) | 2, CCS1377 |
| G3T50_09065 | CP048772 | ZS007 | hypothetical protein (plasmid pCJ145K) | 2, CCS1377 |
| GRN90_01625 | CP047482 | CFSAN096297 | methionyl-tRNA formyltransferase | 2, CCS1377 |
| HX739_05710 | CP058295 | A14a | hypothetical protein | 2, CCS1377 |
| HX739_05715 | CP058295 | A14a | hypothetical protein | 2, CCS1377 |
| MA544_05330 | CP092017 | 15065A | hypothetical protein | 2, CCS1377 |
| MA544_05335 | CP092017 | 15065A | hypothetical protein | 2, CCS1377 |
| NCTC13268_01053 | LR134497 | NCTC13268 | uncharacterised protein | 2, CCS1377 |
| NCTC13268_01054 | LR134497 | NCTC13268 | uncharacterised protein | 2, CCS1377 |
| NCTC13268_01055 | LR134497 | NCTC13268 | uncharacterised protein | 2, CCS1377 |
| NCTC13268_01056 | LR134497 | NCTC13268 | uncharacterised protein | 2, CCS1377 |
| NCTC13268_01057 | LR134497 | NCTC13268 | uncharacterised protein | 2, CCS1377 |
| A0056_003050 | CP059375 | 2012D-9520 | hypothetical protein | 1, 2, 3, *C. jejuni*, CCS1377 |
| GRN90_01620 | CP047482 | CFSAN096297 | UDP-4-amino-4,6-dideoxy-N-acetyl-beta-L-altrosamine N-acetyltransferase PseH | 1, 2, 3, *C. jejuni*, CCS1377 |
| FORC83_0171 | CP028933 | FORC_083 | outer membrane siderophore receptor (TonB-dependent) | 1, 2, 3, *C. jejuni*, CCS1377 |
| CRM97_02835 | CP023866 | FDAARGOS_421 | citrate transporter | 2, 3, *C. jejuni* |
| NCTC13255_00040 | LR134499 | NCTC13255 | gamma-glutamyltransferase ggt | 2, 3, *C. jejuni* |
| AEI25_00045 | CP012242 | CJ055CCUA | cytochrome C | 3, *C. jejuni* |
| NCTC11951_01697 | LR134359 | NCTC11951 | Cytochrome c family protein | 3, *C. jejuni* |
| G3T47_02930 | CP048761 | ZS004 | amidohydrolase family protein | 3, *C. jejuni* |
| QMK10_03040 | CP125388 | SKBC25 | SLC13 family permease | 3, *C. jejuni* |
| CJ12661_1487 | CP028911 | NCTC 12661 | anaerobic DMSO reductase DmsABC, chain C, anchor subunit, DmsC | 3, *C. jejuni* |
| CJ12661_1488 | CP028911 | NCTC 12661 | putative dimethyl sulfoxide reductase chaperone DmsD | 3, *C. jejuni* |
| B10089_07450 | AP028330 | BCH-10089 | hypothetical protein | 3, *C. jejuni* |
| P3245_00145 | CP120236 | PS_38_2021 | hypothetical protein | 3 |
| P3245_00140 | CP120236 | PS_38_2021 | molybdopterin-dependent oxidoreductase | 3 |

**Supplementary Table 2. *C. lari* genes**

| **locus tag** | **GenBank accession** | **strain** | **putative protein function** | **clades** |
| --- | --- | --- | --- | --- |
| AAH949_02240 (WJ046_00840) | CP155620 (CP149498) | CCS1377 (NCTC 11352) | 6-hydroxymethylpterin diphosphokinase MptE-like protein | 1, 2, 3 |
| HW242_01185 | CP063088 | 2014D-0218 | hypothetical protein | 1, 2, 3 |
| HW242_01190 | CP063088 | 2014D-0218 | hypothetical protein | 1, 2, 3 |
| HW242_01195 | CP063088 | 2014D-0218 | hypothetical protein | 1, 2, 3 |
| HW242_02360 | CP063088 | 2014D-0218 | hypothetical protein | 1, 2, 3 |
| HW242_02620 | CP063088 | 2014D-0218 | hypothetical protein | 1, 2, 3 |
| HW242_02625 | CP063088 | 2014D-0218 | hypothetical protein | 1, 2, 3 |
| UPTC3659_0956 | CP007775 | NCTC 11845 | hypothetical protein | 1, 2, 3 |
| UPTC3659_0957 | CP007775 | NCTC 11845 | hypothetical protein | 1, 2, 3 |
| UPTC3659_0973 | CP007775 | NCTC 11845 | hypothetical protein | 1, 2, 3 |
| UPTC3659_0974 | CP007775 | NCTC 11845 | hypothetical protein | 1, 2, 3 |
| UPTC3659_0977 | CP007775 | NCTC 11845 | hypothetical protein | 1, 2, 3 |
| UPTC3659_0978 | CP007775 | NCTC 11845 | hypothetical protein | 1, 2, 3 |
| UPTC3659_0989 | CP007775 | NCTC 11845 | hypothetical protein | 1, 2, 3 |
| UPTC3659_0990 | CP007775 | NCTC 11845 | hypothetical protein | 1, 2, 3 |
| UPTC3659_1008 | CP007775 | NCTC 11845 | hypothetical protein | 1, 2, 3 |
| UPTC3659_0982 | CP007775 | NCTC 11845 | putative lipase | 1, 2 |
| HW242_02700 | CP063088 | 2014D-0218 | hypothetical protein | 1, 2 |
| HW242_01180 | CP063088 | 2014D-0218 | hypothetical protein | 2 |
| HW242_02365 | CP063088 | 2014D-0218 | hypothetical protein | 2 |
| UPTC3659_0943 | CP007775 | NCTC 11845 | hypothetical protein | 2 |
| UPTC3659_0964 | CP007775 | NCTC 11845 | hypothetical protein | 2 |
| UPTC3659_0965 | CP007775 | NCTC 11845 | hypothetical protein | 2 |
| UPTC3659_0983 | CP007775 | NCTC 11845 | hypothetical protein | 2 |
| UPTC3659_0991 | CP007775 | NCTC 11845 | hypothetical protein | 2 |
| UPTC3659_1081 | CP007775 | NCTC 11845 | hypothetical protein | 2 |
| UPTC4110_0843 | CP007775 | NCTC 11845 | hypothetical protein | 2 |
| CONCH_0470 | CP007771 | LMG 11760 | hypothetical protein | 2 |
| CONCH_1398 | CP007771 | LMG 11760 | putative membrane protein | 2 |
| HW242_01115 | CP063088 | 2014D-0218 | hypothetical protein | 2 |
| UPTC3659_0948 | CP007775 | NCTC 11845 | hypothetical protein | 2 |
| UPTC3659_0966 | CP007775 | NCTC 11845 | hypothetical protein | 2 |
| UPTC3659_0984 | CP007775 | NCTC 11845 | hypothetical protein | 2 |
| UPTC3659_0985 | CP007775 | NCTC 11845 | hypothetical protein | 2 |
| UPTC3659_1080 | CP007775 | NCTC 11845 | hypothetical protein | 2 |
| UPTC4110_0844 | CP007776 | CCUG 22395 | putative reverse transcriptase | 2 |
| CLCT_0341 | CP043426 | LMG 21009 | ATP-binding protein (AAA domain) | 2, 3 |
| CD56_04565 | CP011372 | Slaughter Beach | hypothetical protein | 2, 3, *C. jejuni* |
| UPTC4110_1217 | CP007776 | CCUG 22395 | membrane bound O-acyl transferase MBOAT family | 2, 3 |
| UPTC4110_0243 | CP007776 | CCUG 22395 | CRISPR/Cas system-associated endoribonuclease Cas2 type II NMENI | 2, 3, *C. jejuni* |
| CD56_04570 | CP011372 | Slaughter Beach | hypothetical protein | 2, 3 |
| CD56_04575 | CP011372 | Slaughter Beach | hypothetical protein | 2, 3 |
| CONCH_0819 | CP007771 | LMG 11760 | hypothetical protein SIR2 domain | 2, 3 |
| UPTC16712_1242 | CP007778 | RM16712 | beta-1,4-N-acetylgalactosaminyltransferase cgtA | 2, 3 |
| UPTC16712_1245 | CP007778 | RM16712 | glycosyltransferase family 2 | 2, 3 |
| UPTC3659_0779 | CP007775 | NCTC 11845 | hemagglutinin domain-containing protein | 2, 3 |
| CD56_05990 | CP011372 | Slaughter Beach | membrane protein | 2, 3, *C. jejuni* |
| HW242_06065 | CP063088 | 2014D-0218 | ShlB/FhaC/HecB family hemolysin / secretion activation protein | 2, 3, *C. jejuni* |
| HW242_01610 | CP063088 | 2014D-0218 | ShlB/FhaC/HecB family hemolysin / secretion activation protein | 2, 3, *C. jejuni* |
| CD56_03810 | CP011372 | Slaughter Beach | hypothetical protein | 2, 3, *C. jejuni* |
| CLCT_1123 | CP043426 | LMG 21009 | hypothetical protein | 3, *C. jejuni* |
| HW242_01605 | CP063088 | 2014D-0218 | filamentous hemagglutinin N-terminal domain-containing protein | 3 |

**Supplementary Table 3. *Campylobacter* virulence-associated genes**

| **locus tag** | **GenBank accession** | **strain** | **putative protein function** | **clades** |
| --- | --- | --- | --- | --- |
| Cj1478c | AL111168 | NCTC 11168 | outer membrane fibronectin-binding protein CadF | 1, 2, 3, *C. jejuni* |
| CJJ81176_1002 | CP000538 | 81–176 | surface-exposed lipoprotein JlpA | 1, 2, 3, *C. jejuni* |
| Cj1349c | AL111168 | NCTC 11168 | putative fibronectin/fibrinogen-binding protein Fnbp | 1, 2, 3, *C. jejuni* |
| Cj1259 | AL111168 | NCTC 11168 | major outer membrane protein MOMP (PorA) | 1, 2, 3, *C. jejuni* |
| Cj1152c | AL111168 | NCTC 11168 | D,D-heptose 1,7-bisphosphate phosphatase GmhB | 1, 2, 3, *C. jejuni* |
| Cj1151c | AL111168 | NCTC 11168 | ADP-glyceromanno-heptose 6-epimerase HldD | 1, 2, 3, *C. jejuni* |
| Cj1150c | AL111168 | NCTC 11168 | D-beta-D-heptose 7-phosphate kinase/D-beta-D-heptose 1-phosphate adenylyltransferase HldE | 1, 2, 3, *C. jejuni* |
| Cj1149c | AL111168 | NCTC 11168 | sedoheptulose 7-phosphate isomerase GmhA | 1, 2, 3, *C. jejuni* |
| Cj1148 | AL111168 | NCTC 11168 | heptosyltransferase II WaaF | 1, 2, 3, *C. jejuni* |
| Cj1146c | AL111168 | NCTC 11168 | putative glucosyltransferase WaaV | 1, 2, 3, *C. jejuni* |
| Cj1143 | AL111168 | NCTC 11168 | two-domain bifunctional protein (beta-1,4-N-acetylgalactosaminyltransferase / CMP-Neu5Ac synthase NeuA1/CgtA | *C. jejuni* |
| Cj1142 | AL111168 | NCTC 11168 | putative UDP-N-acetylglucosamine 2-epimerase NeuC1 | 2, 3, *C. jejuni* |
| Cj1141 | AL111168 | NCTC 11168 | sialic acid synthase (N-acetylneuraminic acid synthetase) NeuB1 | 2, 3, *C. jejuni* |
| Cj1140 | AL111168 | NCTC 11168 | alpha-2,3 sialyltransferase CstIII | *C. jejuni* |
| CJJ81176_1157 | CP000538 | 81–176 | alpha-2,3-sialyltransferase CstII | *C. jejuni* |
| Cj1139c | AL111168 | NCTC 11168 | beta-1,3 galactosyltransferase WlaN | *C. jejuni* |
| Cj1138 | AL111168 | NCTC 11168 | putative glycosyltransferase (LOS synthesis) | 2, 3, *C. jejuni* |
| Cj1137c | AL111168 | NCTC 11168 | putative glycosyltransferase (LOS synthesis) | 3, *C. jejuni* |
| Cj1136 | AL111168 | NCTC 11168 | putative glycosyltransferase (LOS synthesis) | 2, 3, *C. jejuni* |
| Cj1135 | AL111168 | NCTC 11168 | putative two-domain glucosyltransferase (LOS synthesis) | 1, 2, 3, *C. jejuni* |
| Cj1134 | AL111168 | NCTC 11168 | putative lipid A biosynthesis lauroyl acyltransferase HtrB | 1, 2, 3, *C. jejuni* |
| Cj1133 | AL111168 | NCTC 11168 | heptosyltransferase I WaaC | 1, 2, 3, *C. jejuni* |
| Cj1132c | AL111168 | NCTC 11168 | Polysaccharide biosynthesis protein WlaX | 1, 2, 3, *C. jejuni* |
| Cj1131c | AL111168 | NCTC 11168 | UDP-GlcNAc/Glc 4-epimerase GalE | 1, 2, 3, *C. jejuni* |
| Cj1130c | AL111168 | NCTC 11168 | flippase PglK | 1, 2, 3, *C. jejuni* |
| Cj1129c | AL111168 | NCTC 11168 | GalNAc transferase/polymerase PglH | 1, 2, 3, *C. jejuni* |
| Cj1128c | AL111168 | NCTC 11168 | glucosyl transferase PglI | 1, 2, 3, *C. jejuni* |
| Cj1127c | AL111168 | NCTC 11168 | GalNAc transferase PglJ | 1, 2, 3, *C. jejuni* |
| Cj1126c | AL111168 | NCTC 11168 | oligosaccharide transferase to N-glycosylate proteins PglB | 1, 2, 3, *C. jejuni* |
| Cj1125c | AL111168 | NCTC 11168 | GalNAc transferase PglA | 1, 2, 3, *C. jejuni* |
| Cj1124c | AL111168 | NCTC 11168 | galactosyltransferase PglC | 1, 2, 3, *C. jejuni* |
| Cj1123c | AL111168 | NCTC 11168 | acetyltransferase PglD | 1, 2, 3, *C. jejuni* |
| Cj1122c | AL111168 | NCTC 11168 | putative integral membrane protein WlaJ | 2, *C. jejuni* |
| Cj1121c | AL111168 | NCTC 11168 | UDP-4-keto-6-deoxy-GlcNAc C4 aminotransferase PglE | 1, 2, 3, *C. jejuni* |
| Cj1120c | AL111168 | NCTC 11168 | UDP-GlcNAc C4,6 dehydratase PglF | 1, 2, 3, *C. jejuni* |
| Cj1119c | AL111168 | NCTC 11168 | putative integral membrane protein PglG/WlaM | *C. jejuni* |
| Cj1318 | AL111168 | NCTC 11168 | motility accessory factor Maf1 | 1, 3, *C. jejuni* |
| Cj1317 | AL111168 | NCTC 11168 | Pse synthetase PseI | 1, 2, 3, *C. jejuni* |
| Cj1316c | AL111168 | NCTC 11168 | pseudaminic acid biosynthesis PseA protein PseA | 1, 3, *C. jejuni* |
| Cj1315c | AL111168 | NCTC 11168 | imidazole glycerol phosphate synthase subunit HisH | 1, 3, *C. jejuni* |
| Cj1314c | AL111168 | NCTC 11168 | imidazole glycerol phosphate synthase subunit HisF | 1, 3, *C. jejuni* |
| Cj1313 | AL111168 | NCTC 11168 | N-acetyltransferase specific for PseC product,UDP-4-amino-4,6-dideoxy-beta-L-AltNAc PseH | 1, 2, 3, *C. jejuni* |
| Cj1312 | AL111168 | NCTC 11168 | nucleotidase specific for PseC product,UDP-4-amino-4,6-dideoxy-beta-L-AltNAc PseG | 1, 2, 3, *C. jejuni* |
| Cj1311 | AL111168 | NCTC 11168 | putative acylneuraminate cytidylyltransferase PseF | 1, 2, 3, *C. jejuni* |
| Cj1333 | AL111168 | NCTC 11168 | Maf2 (motility accessory protein 2), PseD protein | 1, 2, 3, *C. jejuni* |
| Cj1319 | AL111168 | NCTC 11168 | GDP-N-acetylglucosamine 4,6-dehydratase [NAD+] LegB | 1, 2, 3, *C. jejuni* |
| Cj1320 | AL111168 | NCTC 11168 | GDP-2-acetamido-2,6-dideoxy-alpha-D-xylo-hexos-4-ulose aminotransferase LegC | 1, 2, 3, *C. jejuni* |
| Cj1321 | AL111168 | NCTC 11168 | UDP-3-O-[3-hydroxymyristoyl] glucosamine N-acyltransferase DegT/LpxD | 1, 2, 3, *C. jejuni* |
| Cj1324 | AL111168 | NCTC 11168 | Legionaminic acid biosynthesis protein PtmG | 1, 2, 3, *C. jejuni* |
| Cj1327 | AL111168 | NCTC 11168 | N-acetylneuraminic acid synthetase NeuB2 | 1, 2, 3, *C. jejuni* |
| Cj1328 | AL111168 | NCTC 11168 | UDP-N-acetylglucosamine 2-epimerase NeuC2 | 1, 2, 3, *C. jejuni* |
| Cj1329 | AL111168 | NCTC 11168 | Glucosamine-1-phosphate guanylyltransferase PtmE | 1, 2, 3, *C. jejuni* |
| Cj1330 | AL111168 | NCTC 11168 | Glutamine–fructose-6-phosphate transaminase (isomerizing), isomerase subunit PtmF | 1, 2, 3, *C. jejuni* |
| Cj1331 | AL111168 | NCTC 11168 | CMP-N,N'-diacetyllegionaminic acid synthase PtmB | 1, 2, 3, *C. jejuni* |
| Cj1332 | AL111168 | NCTC 11168 | Glutamine–fructose-6-phosphate transaminase (isomerizing), glutaminase subunit PtmA | 1, 2, 3, *C. jejuni* |
| Cj1413c | AL111168 | NCTC 11168 | capsule polysaccharide modification protein KpsS | 1, 2, 3, *C. jejuni* |
| Cj1414c | AL111168 | NCTC 11168 | capsule polysaccharide modification protein KpsC | 1, 2, 3, *C. jejuni* |
| Cj1415c | AL111168 | NCTC 11168 | Cytidine diphosphoramidate kinase Cysc | *C. jejuni* |
| Cj1425c | AL111168 | NCTC 11168 | D-glycero-alpha-D-manno-heptose 7-phosphate kinase HddA | *C. jejuni* |
| Cj1423c | AL111168 | NCTC 11168 | D-glycero-D-manno-heptose 1-phosphate guanosyltransferase HddC | *C. jejuni* |
| Cj1439c | AL111168 | NCTC 11168 | UDP-galactopyranose mutase Glf | *C. jejuni* |
| Cj1441c | AL111168 | NCTC 11168 | UDP-glucose 6-dehydrogenase KfiD | (2), (3), *C. jejuni* |
| Cj1443c | AL111168 | NCTC 11168 | D-arabinose 5-phosphate isomerase KpsF | 1, 2, 3, *C. jejuni* |
| Cj1444c | AL111168 | NCTC 11168 | capsule polysaccharide export system periplasmic protein KpsD | 1, 2, 3, *C. jejuni* |
| Cj1445c | AL111168 | NCTC 11168 | capsule polysaccharide export system inner membrane protein KpsE | 1, 2, 3, *C. jejuni* |
| Cj1447c | AL111168 | NCTC 11168 | capsule polysaccharide export ATP-binding protein KpsT | 1, 2, 3, *C. jejuni* |
| Cj1448c | AL111168 | NCTC 11168 | KpsM | 1, 2, 3, *C. jejuni* |
| Cj1339c | AL111168 | NCTC 11168 | flagellin A FlaA | 1, 2, 3, *C. jejuni* |
| Cj1338c | AL111168 | NCTC 11168 | flagellin B FlaB | 1, 2, 3, *C. jejuni* |
| Cj0720c | AL111168 | NCTC 11168 | flagellin C FlaC | 1, 2, 3, *C. jejuni* |
| Cj0337c | AL111168 | NCTC 11168 | flagellar motor proton channel motA | 1, 2, 3, *C. jejuni* |
| Cj0336c | AL111168 | NCTC 11168 | flagellar motor protein MotB | 1, 2, 3, *C. jejuni* |
| Cj0041 | AL111168 | NCTC 11168 | flagellar hook-length control protein FliK | 1, 2, 3, *C. jejuni* |
| Cj0042 | AL111168 | NCTC 11168 | flagellar hook assembly protein FlgD | 1, 2, 3, *C. jejuni* |
| Cj0043 | AL111168 | NCTC 11168 | flagellar hook protein FlgE | 1, 2, 3, *C. jejuni* |
| Cj0351 | AL111168 | NCTC 11168 | flagellar motor switch protein FliN | 1, 2, 3, *C. jejuni* |
| Cj0060c | AL111168 | NCTC 11168 | flagellar motor switch protein FliM | 1, 2, 3, *C. jejuni* |
| Cj0063c | AL111168 | NCTC 11168 | Flagellar synthesis regulator FleN | 1, 2, 3, *C. jejuni* |
| Cj0064c | AL111168 | NCTC 11168 | flagellar biosynthesis protein FlhF | 1, 2, 3, *C. jejuni* |
| Cj0195 | AL111168 | NCTC 11168 | flagellum-specific ATP synthase FliI | 1, 2, 3, *C. jejuni* |
| Cj0318 | AL111168 | NCTC 11168 | flagellar M-ring protein FliF | 1, 2, 3, *C. jejuni* |
| Cj0319 | AL111168 | NCTC 11168 | flagellar motor switch protein FliG | 1, 2, 3, *C. jejuni* |
| Cj0320 | AL111168 | NCTC 11168 | flagellar assembly protein FliH | 1, 2, 3, *C. jejuni* |
| Cj0335 | AL111168 | NCTC 11168 | flagellar biosynthetic protein FlhB | 1, 2, 3, *C. jejuni* |
| Cj0687c | AL111168 | NCTC 11168 | flagellar L-ring protein precursor FlgH | 1, 2, 3, *C. jejuni* |
| Cj0882c | AL111168 | NCTC 11168 | flagellar biosynthesis protein FlhA | 1, 2, 3, *C. jejuni* |
| Cj1179c | AL111168 | NCTC 11168 | flagellar biosynthetic protein FliR | 1, 2, 3, *C. jejuni* |
| Cj0284c | AL111168 | NCTC 11168 | chemotaxis histidine kinase CheA | 1, 2, 3, *C. jejuni* |
| Cj0924c | AL111168 | NCTC 11168 | MCP protein-glutamate methylesterase CheB | 1, 2, 3, *C. jejuni* |
| Cj0923c | AL111168 | NCTC 11168 | MCP protein methyltransferase CheR | 1, 2, 3, *C. jejuni* |
| Cj0285c | AL111168 | NCTC 11168 | Chemotaxis protein CheV | 1, 2, 3, *C. jejuni* |
| Cj0283c | AL111168 | NCTC 11168 | Positive regulator of CheA protein activity CheW | 1, 2, 3, *C. jejuni* |
| Cj1118c | AL111168 | NCTC 11168 | chemotaxis regulatory protein CheY | 1, 2, 3, *C. jejuni* |
| Cj0914c | AL111168 | NCTC 11168 | Campylobacter invasion protein B CiaB | 1, 2, 3, *C. jejuni* |
| Cj1242 | AL111168 | NCTC 11168 | Campylobacter invasion protein C ciaC | 1, 2, 3, *C. jejuni* |
| Cj0788 | AL111168 | NCTC 11168 | Campylobacter invasion protein D CiaD | 1, 2, 3, *C. jejuni* |
| Cj1450 | AL111168 | NCTC 11168 | Campylobacter invasion protein I (ATP/GTP-binding protein) CiaI | 1, 2, 3, *C. jejuni* |
| Cj0079c | AL111168 | NCTC 11168 | cytolethal distending toxin A CdtA | *C. jejuni* |
| Cj0078c | AL111168 | NCTC 11168 | cytolethal distending toxin B CdtB | 1, 2, 3, *C. jejuni* |
| Cj0077c | AL111168 | NCTC 11168 | cytolethal distending toxin C CdtC | *C. jejuni* |
| Cj0979c | AL111168 | NCTC 11168 | putative secreted nuclease CjeN | 1, 2, 3, *C. jejuni* |
| Cj0780 | AL111168 | NCTC 11168 | periplasmic nitrate reductase NapA | 1, 2, 3, *C. jejuni* |
| Cj0588 | AL111168 | NCTC 11168 | putative haemolysin TlyA | 1, 2, 3, *C. jejuni* |
| Cj0175c | AL111168 | NCTC 11168 | putative iron-uptake ABC transport system, periplasmic iron-binding protein CfbpA | 1, 2, 3, *C. jejuni* |
| Cj0174c | AL111168 | NCTC 11168 | putative iron-uptake ABC transport system permease protein CfbpB | 1, 2, 3, *C. jejuni* |
| Cj0173c | AL111168 | NCTC 11168 | putative iron-uptake ABC transport system ATP-binding protein CfbpC | 1, 2, 3, *C. jejuni* |
| Cj0367c | AL111168 | NCTC 11168 | periplasmic fusion protein CmeA (multidrug efflux system CmeABC) | 1, 2, 3, *C. jejuni* |
| Cj0366c | AL111168 | NCTC 11168 | inner membrane efflux transporter CmeB (multidrug efflux system CmeABC) | 1, 2, 3, *C. jejuni* |
| Cj0365c | AL111168 | NCTC 11168 | outer membrane channel protein CmeC (multidrug efflux system CmeABC) | 1, 2, 3, *C. jejuni* |
| Cj1031 | AL111168 | NCTC 11168 | outer membrane component of efflux system CmeD (multidrug efflux system CmeDEF) | 1, 2, 3, *C. jejuni* |
| Cj1032 | AL111168 | NCTC 11168 | membrane fusion component of efflux system CmeE (mutlidrug efflux system CmeDEF) | 1, 2, 3, *C. jejuni* |
| Cj1033 | AL111168 | NCTC 11168 | integral membrane component of efflux system CmeF (multidrug efflux system CmeDEF) | 1, 2, 3, *C. jejuni* |
| Cj0368c | AL111168 | NCTC 11168 | transcriptional regulator CmeR | 1, 2, 3, *C. jejuni* |
| Cj0643 | AL111168 | NCTC 11168 | two-component response regulator CbrR | 1, 2, 3, *C. jejuni* |
| Cj0921c | AL111168 | NCTC 11168 | aspartate/glutamate-binding ABC transporter protein Peb1/PebA | 1, 2, 3, *C. jejuni* |
| Cj0778 | AL111168 | NCTC 11168 | major antigenic peptide PEB2 | 1, 2, 3, *C. jejuni* |
| Cj0289c | AL111168 | NCTC 11168 | major antigenic peptide PEB3 | 3, *C. jejuni* |
| Cj0596 | AL111168 | NCTC 11168 | major antigenic peptide PEB4-cell binding factor | 1, 2, 3, *C. jejuni* |
| Cj0922c | AL111168 | NCTC 11168 | ABC-type amino-acid transporter ATP-binding protein pebC | 1, 2, 3, *C. jejuni* |
| Cj0920c | AL111168 | NCTC 11168 | putative ABC-type amino-acid transporter permease protein PebE | 1, 2, 3, *C. jejuni* |
| Cj0919c | AL111168 | NCTC 11168 | putative ABC-type amino-acid transporter permease protein PebF | 1, 2, 3, *C. jejuni* |
| CJJ81176_0067 | CP000538 | 81–176 | gamma-glutamyltransferase *ggt* | 2, 3, *C. jejuni* |
| CJJ81176_0056 | CP000538 | 81–176 | L-asparaginase AnsB | 1, 2, 3, *C. jejuni* |
| CJJ81176_1570 | CP000538 | 81–176 | anaerobic dimethyl sulfoxide reductase chain A DmsA | 3, *C. jejuni* |
| CJJ81176_1571 | CP000538 | 81–176 | anaerobic dimethylsulfoxide reductase, chain B DmsB | 3, *C. jejuni* |
| CJJ81176_1572 | CP000538 | 81–176 | anaerobic dimethylsulfoxide reductase, chain C DmsC | 3, *C. jejuni* |
| CJJ81176_1573 | CP000538 | 81–176 | anaerobic dimethylsulfoxide reductase, chain D DmsD | 3, *C. jejuni* |
| Cj1228c | AL111168 | NCTC 11168 | serine protease (protease DO) HtrA | 1, 2, 3, *C. jejuni* |
| Cj0486 | AL111168 | NCTC 11168 | L-fucose permease | 1, *C. jejuni* |
| Cj1385 | AL111168 | NCTC 11168 | Catalase katA | 1, 2, 3, *C. jejuni* |
| Cj0169 | AL111168 | NCTC 11168 | superoxide dismutase sodB | 1, 2, 3, *C. jejuni* |
| A17_01900 | CP028373 | huA17 | type VI secretion system protein VgrG (TssI | 2, *C. jejuni* |
| A17_01901 | CP028373 | huA17 | type VI secretion protein ImpH/VasB (TssG) | 2, *C. jejuni* |
| A17_01902 | CP028373 | huA17 | type VI secretion protein ImpG/VasA (TssF) | 2, *C. jejuni* |
| A17_01903 | CP028373 | huA17 | type VI secretion system lysozyme-like protein Vca0109(TssE/gp25) | 2, *C. jejuni* |
| A17_01904 | CP028373 | huA17 | type VI secretion protein ImpC (TssC/VipB) | 2, *C. jejuni* |
| A17_01905 | CP028373 | huA17 | type VI secretion protein ImpB (TssB/VipA) | 2, *C. jejuni* |
| A17_01906 | CP028373 | huA17 | type VI secretion-associated protein ImpA(TssA/VasJ) | 2, *C. jejuni* |
| A17_01907 | CP028373 | huA17 | type VI secretion lipoprotein VasD (TssJ) | 2, *C. jejuni* |
| A17_01908 | CP028373 | huA17 | type VI secretion protein ImpJ/VasE (TssK) | 2, *C. jejuni* |
| A17_01909 | CP028373 | huA17 | type IV/VI secretion system protein ImpK/VasF(TssL), OmpA/MotB domain | 2, *C. jejuni* |
| A17_01910 | CP028373 | huA17 | Secreted protein , hemolyis co-regulated protein hcp (TssD) | 2, *C. jejuni* |
| A17_01911 | CP028373 | huA17 | type VI secretion protein IcmF (TssM) | 2, *C. jejuni* |
| CJJ81176_RS08620 | NC_008770 | 81–176 pVir | putative type IV secretion protein; also competence Protein VirB8 | *C. jejuni* |
| CJJ81176_RS08625 | NC_008770 | 81–176 pVir | TrbG/VirB9 family P-type conjugative transfer protein | *C. jejuni* |
| CJJ81176_RS08630 | NC_008770 | 81–176 pVir | VirB10 putative type IV secretion protein, competence protein | *C. jejuni* |
| CJJ81176_RS08640 | NC_008770 | 81–176 pVir | CpaF/VirB11 family protein | *C. jejuni* |
| CJJ81176_RS08650 | NC_008770 | 81–176 pVir | putative type IV secretion protein; similar to Helicobacter pylori VirD4 (traG conjugation protein) | *C. jejuni* |
| CJJ81176_RS08665 | NC_008770 | 81–176 pVir | Inner membrane protein of type IV secretion of T-DNA complex, VirB6 | *C. jejuni* |
| CJJ81176_RS08855 | NC_008770 | 81–176 pVir | ATPase required for both assembly of type IV secretion complex and secretion of T-DNA complex, VirB4 | *C. jejuni* |
| CJJ81176_RS09190 | NC_008770 | 81–176 pVir | type IV secretion system protein VirB7 | *C. jejuni* |

**Supplementary Table 4. *Campylobacter* antimicrobial resistance genes**

| **locus tag** | **GenBank accession** | **strain** | **gene / putative protein function** | **antimicrobial** |
| --- | --- | --- | --- | --- |
| ATE51_03620 | CP013733 | *C. coli* OR12 | *bla*_OXA−489_ | penicillin / ampicillin |
| - | WP_002857904 | *Campylobacter* sp. | *gyrA* / DNA topoisomerase (ATP-hydrolyzing) subunit A (WT vs S22G/T86I/T665S) | quinolones |
| - | MN461240 | *C. coli* SM17 | *ermB* / rRNA adenine-N-6-methyltransferase | macrolides |
| ATE51_00064 | CP013733 | *C. coli* OR12 | 23S ribosomal RNA (WT vs A2075G) | macrolides |
| BHT78_06985 | EAK3643206 | *C. coli* | *rplD* 50S ribosomal protein L4 (WT vs V82I, T91K, V121A, V176I, T177S, M192I) | macrolides |
| G3T50_07595 | CP048771 | *C. jejuni*  ZS007 | *cat* type A-13, cloramphenicol-acetyltransferase | cloramphenicol |
| AAID91_01225 | CP154494 | *C. coli* CCS 551 | CatA-like O-acetyltransferase, cloramphenicol-acetyltransferase | cloramphenicol |
| G3R55_07255 | CP048769 | *C. jejuni* ZJB020 | *fexA* chloramphenicol/florfenicol efflux MFS | cloramphenicol |
| NQK33_03735 | CP103055 | *C. coli* YC3-24 | *tetL*, tetracycline efflux MFS transporter Tet(L) | tetracycline |
| CFK74_08920 | CP022471 | *C. jejuni* RM1246 | *tetO*, tetracycline resistance ribosomal protection protein Tet(O) | tetracycline |
| AAH949_08810 | CP155620 | *Campylobacter* sp. CCS1377 | *aadE*-Cc, aminoglycoside 6-adenylyltransferase | aminoglycosides |
| - | MT176413 | *C. jejuni* CITCj625-18 | *ant*(6)-Ib, aminoglycoside 6-nucleotidyltransferase protein Ib | aminoglycosides |
| AAID97_02465 | CP154432 | *C. coli* strain CCS 1744/23 | *aac*(3), AAC(3) family N-acetyltransferase | aminoglycosides |
| AAID97_01080 | CP154432 | *C. coli* strain CCS 1744/23 | *ant*(6)-Ig, aminoglycoside nucleotidyltransferase ANT(6)-Ig | aminoglycosides |
| AAID97_01165 | CP154432 | *C. coli* strain CCS 1744/23 | *aac*(6'), aminoglycoside 6'-N-acetyltransferase | aminoglycosides |
| O8035_03025 | CP115181 | *C. coli* 18JS15S | *aph*(2'')-Ia, aminoglycoside O-phosphotransferase APH(2'')-Ia | aminoglycosides |
| G3R55_00365 | CP048769 | *C. jejuni* ZJB020 | *aph*(2'')-If, aminoglycoside O-phosphotransferase APH(2'')-If | aminoglycosides |
| G3T45_03570 | CP048764 | *C. jejuni* ZH003 | *aph*(3')-IIIa, aminoglycoside O-phosphotransferase APH(3')-IIIa | aminoglycosides |
| - | EAJ3704114 | *C. coli* | RpsL 30S ribosomal protein S12 (WT vs K43R) | streptomycin |
| O8035_03015 | CP115181 | 18JS15S | *sat4*, streptothricin N-acetyltransferase Sat4 | streptothricin |

**Supplementary Table 5.** Susceptibility testing results (inhibition zone diameter in mm)

| isolate | clade | Penicillin P10 | Ampicillin AMP10 | Cefazolin CZ30C | Cefotaxime CTX30 | Ciprofloxacin CIP5 | Erythromycin E15 | Tetracycline TE30 | Chloramphenicol C30 | Florphenicol FFC30 | Gentamicin CN10 | Streptomycin S25 | Kanamycin K5 |
| --- | --- | --- | --- | --- | --- | --- | --- | --- | --- | --- | --- | --- | --- |
| CCS 24982/43 | 2 | 13 | 31 | 6 | 35 | 40 | 40 | 40 | 34 | 47 | 22 | 31 | 13 |
| CCS 17343/22 | 2 | 10 | 24 | 6 | 32 | 46 | 44 | 44 | 36 | 50 | 25 | 15 | 11 |
| CCS 07/803 | 2 | 6 | 18 | 6 | 32 | 46 | 42 | 40 | 40 | 50 | 25 | 21 | 12 |
| CCS 1744/23 | 2 | 13 | 26 | 6 | 31 | 46 | 32 | 40 | 38 | 48 | 26 | 20 | 16 |
| CCS 249 07/13784 | 2 | 11 | 29 | 6 | 32 | 40 | 35 | 40 | 34 | 47 | 24 | 31 | 12 |
| CCS 554 | 2 | 12 | 23 | 6 | 27 | 44 | 37 | 44 | 40 | 47 | 25 | 30 | 13 |
| CCS 551 | 2 | 8 | 28 | 6 | 32 | 42 | 36 | 40 | 35 | 50 | 22 | 29 | 12 |
| CCS 17341/20 | 3 | 17 | 30 | 6 | 32 | 40 | 38 | 40 | 37 | 45 | 25 | 30 | 14 |
| CCS 265 07/14005 | 3 | 12 | 25 | 6 | 31 | 40 | 34 | 40 | 40 | 48 | 26 | 32 | 15 |
| CCS 289 08/401 | 3 | 9 | 17 | 6 | 27 | 40 | 28 | 37 | 33 | 40 | 21 | 25 | 12 |
| BFR-CA-9557 | 1 | 6 | 22 | 6 | 18 | 6 | 30 | 37 | 38 | 40 | 23 | 30 | 11 |
| RM2228 | 1 | 10 | 22 | 6 | 22 | 34 | 6 | 6 | 35 | 45 | 25 | 30 | 6 |
| NCTC 11168 | n.a. | 15 | 22 | 6 | 34 | 35 | 36 | 42 | 40 | 46 | 24 | 30 | 16 |
| 81–176 | n.a. | 10 | 20 | 6 | 20 | 40 | 36 | 40 | 42 | 45 | 26 | 31 | 10 |
| CCS 1377 | n.a. | 6 | 14 | 6 | 30 | 33 | 32 | 40 | 35 | 40 | 22 | 26 | 6 |
